# A framework for the extended monitoring of levels of cognitive function in unresponsive patients

**DOI:** 10.1371/journal.pone.0200793

**Published:** 2018-07-19

**Authors:** Richard L. Mah, John F. Connolly

**Affiliations:** 1 Department of Linguistics and Languages, McMaster University, Hamilton, Ontario, Canada; 2 Centre for Advanced Research in Experimental and Applied Linguistics, McMaster University, Hamilton, Ontario, Canada; 3 Department of Psychology, Neuroscience and Behaviour, McMaster University, Hamilton, Ontario, Canada; 4 School of Biomedical Engineering, McMaster University, Hamilton, Ontario, Canada; University of British Columbia, CANADA

## Abstract

Generally, prognostication of coma outcome currently combines behavioral, reflex, and possibly neuroimaging tests that are interpreted by an attending physician. Electroencephalography, particularly, event-related brain potentials (ERP) have received attention due to evidence demonstrating the positive predictive value of certain ERP including the mismatch negativity (MMN) and the P3a, for coma emergence. We describe a set of ERP paradigms designed to require and reflect increasing levels of cognitive processing with the added objective of determining the influence of each paradigm’s context strength on its ability to elicit ERPs. These paradigms were then used without explicit instructions to participants to attend to the stimuli to determine which paradigms possessed sufficient context “strength” to elicit ERPs in the absence of active participation on the part of the subject; a circumstance often encountered in brain injury patients. These paradigms were then validated on two groups of adults–younger and older, and the difference due to active participation was validated on another group of younger adults. Results show that paradigms with stronger stimulus context features performed better than those with weaker contexts, and that older adults generally had significantly attenuated and delayed responses compared to younger adults. Based on these findings, it is recommended the use of the auditory oddball paradigm that includes novel stimuli to elicit the mismatch negativity and P300, and semantic violation sentences to elicit the N400. These findings also reinforce the procedure of instructing participants about the requirements of a protocol–regardless of the patient’s diagnosis or apparent state–in order to help those who are able to attend to show the most robust responses possible.

## Introduction

The use of event related potentials (ERPs) for the assessment of patients in altered states of consciousness has been a topic of research for the past few decades. Some of the first prognostic studies involved the use of the P300 in non-traumatic comas, where the presence of the P300 was correlated with positive outcomes [1–3].

Some researchers have taken issue with the use of the P300, which is heavily influenced by the participant attending to the stimuli. The proposed solution was to use an ERP component, which could be evoked irrespective of attention, like the mismatch negativity (MMN). One of the first to do this was Kane et al., who presented tones in an auditory oddball sequence [4]. Since the MMN has been strongly elicited irrespective of attention, they believed it to be a more reliable measure for patients in altered states of consciousness [5, 6]. This study showed that in each case the MMN was detected, the patient would soon regain consciousness. They noted that the “MMN response is the earliest available indicator of awakening from coma” while acknowledging that the MMN “does not provide prognostic information about functional outcome, [but] it may help to define objectively the duration of coma” [4].

Fischer et al. built upon this work by expanding the use of the MMN in comatose patients [7]. These studies, however, utilized the traditional method of ERP analysis that is time consuming, requires specialized tools, and visual inspection by a trained electrophysiologist. Fischer and colleagues used stimuli which were delivered in short blocks, and only those blocks that were visually identified as containing the N100 and P200 were further scrutinized for the MMN [7]. Depending on the quality of the recorded data, this could lead to large quantities of discarded data, which ultimately makes the technique harder for clinicians to use. Despite these methods running the risk of data loss and the requirement of expert examination, they remain the gold standard for much of the clinical research literature [8, 9].

More recent investigations by Tzovara et al. have tried to reduce the quantity of data needed to generate meaningful information regarding the presence or absence of the MMN [10]. Using an automated classification technique to quantify the neural response of each individual instead of an expert visual inspection, they were able to take a whole data set and blindly classify it, thereby increasing the amount of useful data. Furthermore, even though the model was able to accurately classify the neural responses of non-survivors, it was the positive or negative progression over time that was the major predictor of outcomes.

A notable commonality of this literature is the frequent use of greatly different MMN elicitation protocols and analysis methods to confirm its presence. This difference in method may be a contributing factor to the wide range in test specificity seen across studies. The inconsistency in test specificities is one of the reasons preventing clinicians from bringing ERP tests into a health care setting (see Discussion).

The current clinical gold standard of outcome prognosis include the Glasgow Coma Scale (GCS) [11], which relies heavily on the patient’s ability to produce behavioral responses to external stimuli and commands. However, early evoked potentials, like somatosensory responses and brainstem auditory potentials are also of high prognostic value [7, 12]. Tests that utilize later cortical responses like the MMN have further improved the prognostic value of these tests while continuing the move away strictly behaviorally based ones [13]. Such improvement is necessary considering the misdiagnosis rates for the unresponsive wakefulness syndrome (UWS) of between 41% [14] to 43% [15] when relying on traditional behaviorally-based consensus methods. More structured behavioral assessment can significantly improve the diagnosis of UWS [14] and there is every reason to believe that functional brain measures would further improve diagnostic accuracy [16, 17].

Kane suggested that the use of attention-modulated ERP components (e.g., P300) was a hindrance to their prognostic power because the response is larger in attentive individuals and is even absent in some healthy controls [4]. However, it is precisely the sensitivity of certain responses to changes in attention that increases the power of these methods to examine levels of function in disorders of consciousness (DOC) patients and with improved protocols the absence of such responses in controls can be reduced dramatically.

In addition to the P300 reflecting different levels of cognitive activity, it is also sensitive to more sophisticated cognitive process such as memory-based processing including recognition of one’s own name. For example, Holeckova et al. used a classic auditory oddball paradigm comprised of standard and infrequently occurring deviant tone stimuli, but was also able to elicit the P300 in response to rare and more salient novel sounds, such as the Subject’s Own Name (SON) [18]. The SON test is of particular interest as it is known to capture attention in the absence of effort [19] and elicits a robust P300 response [20–23].

Building on this, another component that is sensitive to contextually-based expectancies is the N400. This component requires the integration of both pre-existing knowledge and newly parsed information. It is a late-occurring response associated broadly with comprehension of speech, text, and other stimuli possessing “meaning” [24].

Expectations can be set up through the use of word pairs that may or may not have underlying semantic relationships [25], or sentences with incongruous, unexpected or infrequent endings [26]. With word pairs, the N400 occurs to the second word when it violates the semantic context created by the first word. A significantly stronger semantic context is created by sentences so that a word that fails to meet contextually-based expectations results in a large N400 in contrast to little or no response to a contextually appropriate word [24]. In these cases, the mismatching words must be interpreted within a particular context, the processing of which requires elaborated attention and memory. Within the context of DOC, the evaluation of receptive language functions provides an objective measure of cognitive processing and, by implication, the level of consciousness.

This study addresses these issues by examining the various stimulation paradigms often used in the literature and selecting those that are best able to elicit strong ERP components of interest. The strongest paradigms will be integrated into a framework for extended and repeated testing of patients in comas for the prediction of both coma emergence and functional outcome. The framework’s design allows for data to be collected over extended durations and at milestone points during a patient’s recovery, giving a better understanding of a patient’s recovery trajectory and to further examine the stability of these components in brain injured patients over time. This framework does not prescribe a specific analysis method to replace expert visual inspection but rather aims to generate data that is agnostic toward the method of analysis. The components elicited should be strong enough to be detected using both traditional methods as well as newer machine learning-based methods.

## Methods

### Participants

Two groups of participants were recruited based on age range. Twenty-six native English speaking undergraduate students (19 females) from McMaster University were recruited for the younger adult group. Thirteen native English speaking adults (6 females) from the Hamilton community were recruited for the older adult group. Participants were 18 to 22 and 66 to 76 years old (*M* = 19.8, *SD* = 1.44; *M* = 69.8, *SD* = 3.35) for the younger and older adult groups, respectively. All participants were dextral (*M* = 85.6; Edinburgh Handedness Inventory Laterally Quotient Range: 40–100; [27]), had no history of neurological diseases or disorders, and had normal or corrected-to-normal vision. Undergraduate students received course credit for their participation, and older adults received $20.

A third group of participants was recruited for the follow up study and included twelve native English speaking students (10 females) from McMaster University. These participants were 19 to 25 (*M* = 21.0, *SD* = 1.78), were dextral (*M* = 93.7; Laterally Quotient Range: 40–100; [27]), had no history of neurological diseases or disorders, and had normal or corrected-to-normal vision.

### Electrophysiological methods

The electroencephalogram (EEG) was recorded continuously (bandpass = 0.01–100 Hz and sampled at 512 Hz) using a 64 channel Biosemi ActiveTwo system (Biosemi, Amsterdam, The Netherlands) using a 10-20 elastic cap with Ag/AgCl electrodes. The electrooculogram (EOG) was recorded from electrodes placed above and at the outer canthus of the left eye. References were recorded bilaterally from the mastoids and at the nasion for offline referencing.

Data preprocessing was conducted using BrainVision Analyzer 2. All recordings were visually inspected and epochs containing artifacts (e.g., muscle, movement) removed. Individual subtask recordings were filtered offline with a bandpass of 0.1–30 Hz. Ocular artifacts were corrected using the *Ocular ICA* transformation provided by BrainVision Analyzer 2.

Trials were segmented into epochs depending on the component of interest. Different pre-stimulus intervals for each component of interest were chosen based on the analysis methods used in the original work. For segments containing the MMN: 100 ms pre-stimulus to 500 ms post-stimulus (as in [12]); the P300: 200 ms pre-stimulus to 1000 ms post-stimulus (as in [18]); the N400: 100 ms pre-stimulus to 1000 ms post-stimulus (as in [26]). Segments for each subtask were baseline corrected together to remove mean pre-stimulus activity. A final artifact rejection was performed automatically, removing segments with voltage steps greater than 50μV, voltage differences greater than 200μV in 200 ms, and channels with low activity (<0.5μV). Segments were averaged together per condition, per participant for each subtask.

In each condition, peaks were automatically detected for each channel independently within the following epochs: N1: 110–190 ms, P2: 180–280 ms, MMN: 120–240 ms, P300: 270–450 ms, N400: 300–700 ms. For further analysis, the latency and the mean amplitude of a 50 ms epoch around each peak were determined for each condition and participant.

### Assessment battery

A battery of tasks was developed to evaluate increasing levels of auditory, cognitive, and linguistic processing. As this battery will be used to assess the level of consciousness of comatose patients, participants were informed that the auditory stimuli were of no relevance to the study and were free to view a silent film. The working hypothesis is that comatose patients are incapable of processing environmental stimuli, so the instructions were intended to better approximate the possible variability in the mental state of patients. The third group of participants received additional instructions (see Behavioural manipulation section of Procedure). The total time required to administer these tests was approximately 90 minutes. A brief description of each task follows.

All stimulus items were normalized using WaveGain, which is a program that applies the ReplayGain standard to sound files. The ReplayGain standard is a normalization technique that is based on the perceptual loudness of sounds. Auditory stimulus delivery was calibrated to 89 dB SPL using a continuous 800 Hz tone.

#### Oddball mismatch

The SON protocol [12, 18] has been demonstrated to invoke a P3b component to the subject’s own name. This protocol was combined with an auditory oddball task like that in [12] to which an additional novel sound was included in order to elicit the P3a (c.f. [28]). These protocols were chosen due to their prior use with clinical populations including comatose patients and those with UWS. The modifications introduced in the present study were intended to capture additional but related information on the processing levels already captured by the original protocols.

Stimuli consisted of standard (80%) and deviant (14%) tones, a familiar novel sound (the SON) (3%), and an unfamiliar novel sound that carried no linguistic content (a dog bark) (3%). Tones were digitally generated sine waves of 800 Hz, with a standard tone duration of 75 ms and a deviant tone duration of 30 ms. The familiar novel was a digital recording of the subject’s name spoken by a native speaker of Canadian English in a neutral voice. The unfamiliar novel was a digital recording of a dog barking. Stimuli were presented pseudorandomly (no deviant or novel stimulus was preceded by less than two standard tones) in one block of 2000 items with a stimulus onset asynchrony (SOA) for the tones being 610 ms and 1220 ms for the novels.

#### Pattern violation mismatch

A different MMN-eliciting task was included to determine whether the type of expectancy violation would affect the resulting MMN component. This task was a global pattern violation task (adapted from [29]) and used the same tone stimuli as in the Oddball Mismatch task.

Tones were presented in an alternating pattern (i.e., ABABAB with, for example, A being the longer tone and B the shorter) so that violations were produced when the alternating sequence was altered by the double repetition of one of the stimuli (e.g., ABAB**BB**AB). Stimuli were presented in one block of 2000 items with 8% of the items being first repetition deviants and 8% being second repetition deviants. The stimulus onset asynchrony was 610 ms.

#### Subject’s Own Name (SON)

Similar to the oddball mismatch task above, this task (adapted from [18]) used the subject’s own name to elicit the P300. This task was included to compare the P300 characteristics to names presented within the oddball mismatch to those presented alongside other names and words.

The subject’s first name is presented alongside five other Common First Names (CFN) (two of the same gender as the participant and three of the opposite), and a list of ten mono- or di-syllabic Non-Salient Other Words (NSOW). These non-salient other words were high in frequency and matched the length of the subject’s own name. All words were digital recordings of a speaker of Canadian English reading the words in a neutral voice. Each of these items were presented 60 times for a total of 480 trials. The speaker did not represent a familiar voice or person to the participant.

#### Semantic violation sentences

This task was a replication without behavioral responses of the terminal-word semantic violation paradigm (as in [26]) and used 144 sentences of six to twelve words recorded using natural speech. All sentences were digital recordings of a speaker of Canadian English reading in a neutral voice. These sentences were divided into groups of 36 having either: a semantically congruent terminal word (Phoneme Match-Semantic Match), *He takes his coffee with milk and SUGAR*; a semantically incongruent terminal word (Phoneme Mismatch-Semantic Mismatch), *The pizza was too hot to SING*; a terminal word with low cloze probability (Phoneme Mismatch-Semantic Match), *The pigs wallowed in the PEN*; or a phonological foil having a terminal word that began with the same initial sound as the semantically congruent word (Phoneme Match-Semantic Mismatch), *The gambler had a streak of bad LUGGAGE*.

#### Word-word priming

The final element of the battery was a replication without behavioral responses of the word-word auditory priming task from [25]. All words were recorded using natural speech by a speaker of Canadian English reading in a neutral voice. All word pairs had a valid word of English as a prime. The targets included valid words of English that were either semantically congruent or incongruent, English pseudowords, or noise that was generated by reversing a valid English word. A total of 160 pairs of words were presented with 1150 ms between words and three seconds between trials.

### Procedure

The study was approved by the Hamilton Integrated Research Ethics Board, Hamilton, Ontario, Canada. All persons gave their written informed consent prior to their inclusion in the study, in accordance with the ethical standards of the Declaration of Helsinki. Participants completed a brief demographic questionnaire to ensure they met all inclusion criteria. While seated in a dimly lit room, auditory stimuli were delivered via Etymotic ER-1 insert earphones and participants were instructed to watch a silent video and disregard the sounds occurring in the background. Between each subtest, participants were given a brief break.

#### Behavioral manipulation

The third group of participants were only given a subset of the paradigms in the passive condition, as described above, and then were given the same paradigms with instructions to press buttons depending on the paradigm.

For the SON paradigm, the participants were instructed to press a button when they heard their own name. For the semantic violation sentences paradigm, they were instructed to press one button when the sentence was grammatical, and another if the sentence was ungrammatical. For the word-word priming paradigm, they were instructed to press one button if the target word was a valid English word, and another if the target word was not a valid word of English.

### Statistical analyses

**Fig 1:**
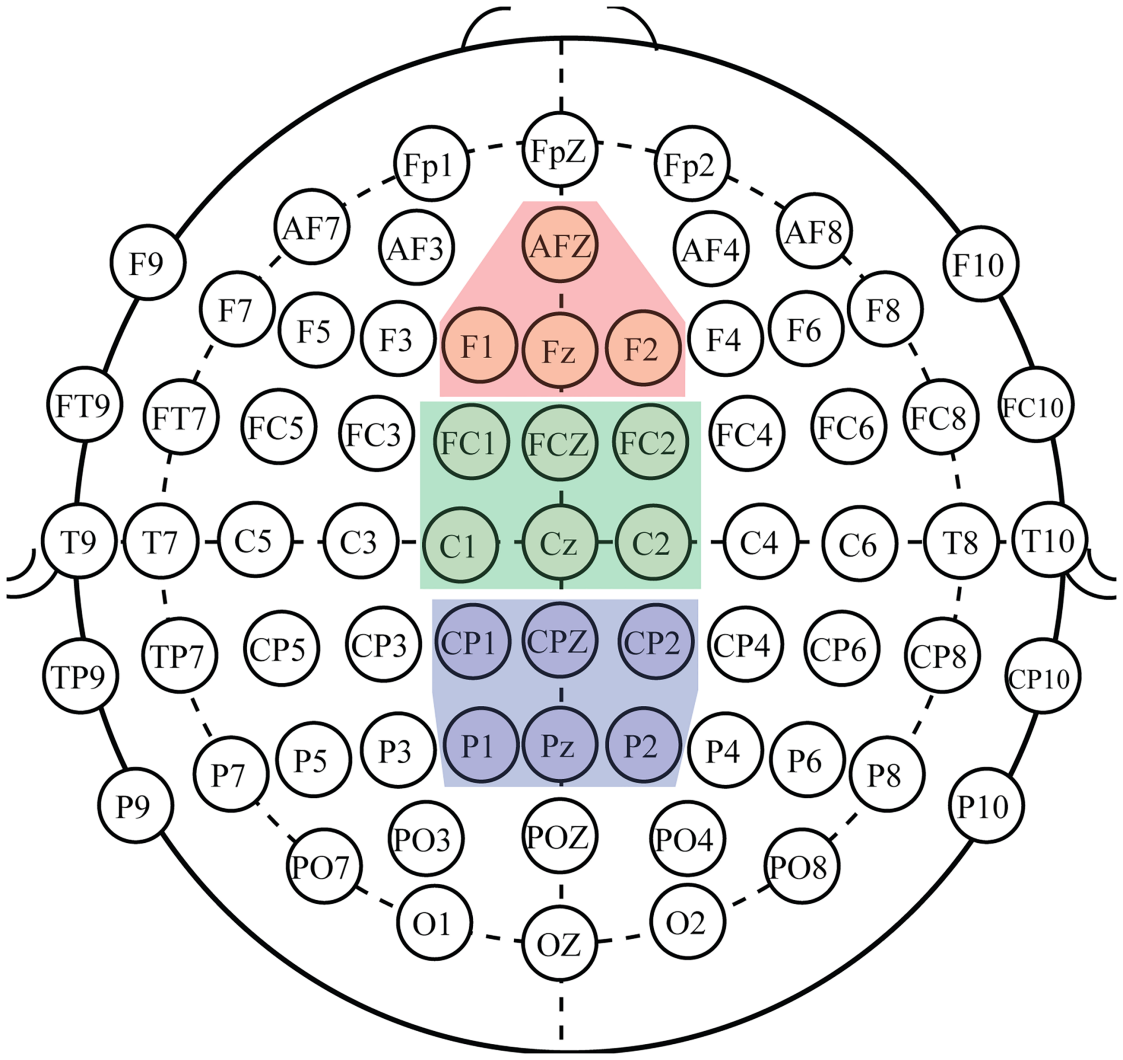
Graphical representation electrode members of the Regions of Interest (ROIs). Mid Frontal (red, 4 electrodes): F1, F2, Fz, AFz. Mid Central (green, 6 electrodes): C1, C2, Cz, FC1, FC2, FCz. Mid Parietal (blue, 6 electrodes): P1, P2, Pz, CP1, CP2, CPz.

The ERP peak amplitude values were extracted by computing the mean value of 50 ms windows centered on the detected ERP peak. ERP peak amplitude and latency data were analyzed using separate mixed-design analysis of variance (ANOVA) models. The ANOVAs were conducted as omnibus tests with Greenhouse-Geisser corrections being applied to the degrees of freedom. All corrected probabilities are reported. Analyses were conducted separately for each detected peak’s amplitude and latency in each subtask individually using *Group* (younger and older adult) as a between-subjects factor, and *Condition* (levels dependent on protocol) and *ROI* (Mid Frontal, Mid Central, and Mid Parietal) as within-subjects factors. The ROIs were defined per the NEMO ROI plan [30], and as shown in Fig 1.

To determine if the MMN was present for the pattern violation mismatch paradigm, the mean MMN peak amplitude for each combination of group, ROI, and deviant was compared for significant differences from zero using a one-tailed t-test [31].

## Results

### Oddball mismatch

#### MMN

**Fig 2:**
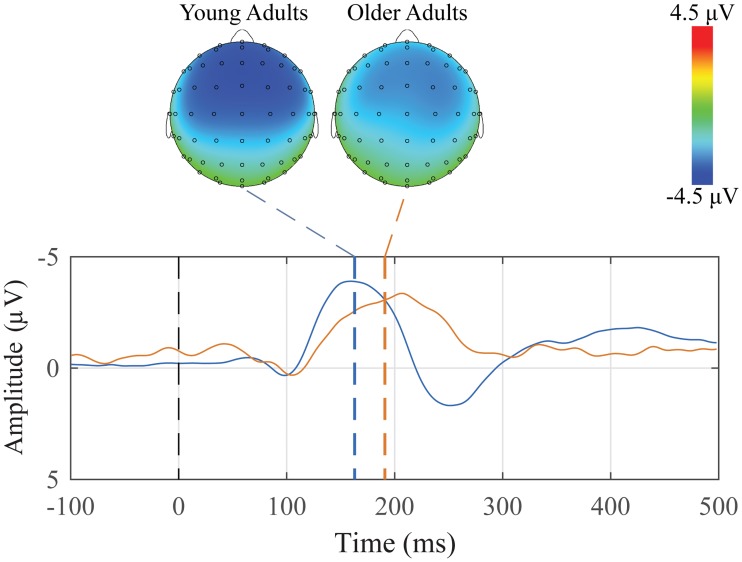
Grand average difference waveforms in the Mid Central ROI of the oddball mismatch MMN and corresponding peak topographic maps. The mean difference response for the younger adult (blue) and older adult (orange) groups are plotted. Dashed colored lines indicate the mean group latency from individually scored MMN peak latencies. Scalp topography maps show voltage distributions at mean group peak latencies.

Grand average subtraction waveforms for both groups with peak topographic maps are presented in Fig 2. For younger adults, 21 of 26 participants showed the oddball mismatch, and for older adults, 12 of 13 participants showed the oddball mismatch.

It is readily apparent that the MMN occurs earlier in the younger adults than in the older adults. It is also clear that waveform morphology differed between the two groups with younger adults exhibiting a small post-MMN positivity (P200) but no such response being seen in older adults. Finally, the MMN topographical maps show a stronger and more focused scalp distribution for younger adults than for older adults.

Descriptive statistics for the amplitude and latency for both groups and the three ROIs are presented in Table 1. Mixed-design ANOVAs were conducted separately for amplitude and latency with *Group* (younger and older adults) as a between-subjects factor and *ROI* (Mid Frontal, Mid Central, and Mid Parietal) as a within-subject factor. Since the MMN is most readily detected by using subtractions between the deviant and standard conditions, there is no *Condition* factor in the ANOVA.

**Table 1:** Means and standard errors of the mean of MMN peak amplitudes and latencies for the oddball mismatch.

ROI	Younger Adults		Older Adults	
	Amplitude (SEM)	Latency (SEM)	Amplitude (SEM)	Latency (SEM)
Mid Frontal	-3.23 (0.20)	158.5 (1.5)	-2.88 (0.16)	186.0 (2.9)
Mid Central	-3.10 (0.14)	163.3 (1.4)	-3.00 (0.10)	191.2 (2.3)
Mid Parietal	-2.37 (0.14)	167.9 (1.7)	-2.19 (0.11)	196.8 (2.2)

There was only a main effect of ROI for amplitude (F(2,74) = 44.001, *p* < 0.001), with both the Mid Central and Mid Frontal ROIs being significantly more negative than the Mid Parietal ROI (all *p*′ *s* < 0.001). There were both main effects of group (F(1,37) = 25.694, *p* < 0.001) and ROI (F(2,74) = 13.443, *p* < 0.001) for latency. The peaks for younger adults were significantly earlier than the older adults, and both the Mid Central and Mid Frontal ROIs occurring significantly earlier than the Mid Parietal ROI (all *p*′ *s* < 0.02).

#### P300 response to novels

**Fig 3:**
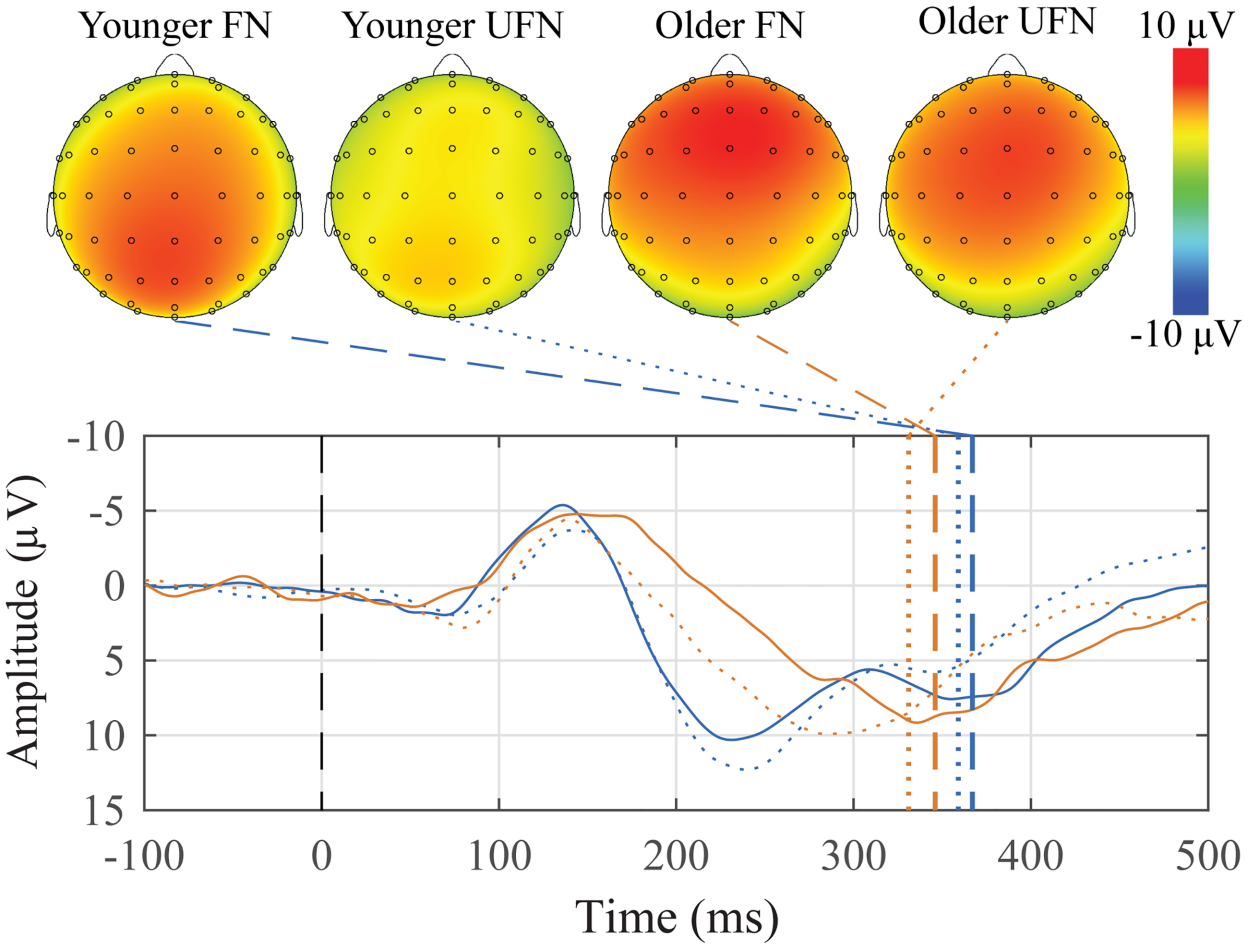
Grand average waveforms at Mid Central ROI to the familiar and unfamiliar novels and corresponding peak topographic maps within the oddball mismatch. The mean responses to the familiar novel (FN) for younger adults (blue) and older adults (green), and the unfamiliar novel (UFN) for younger adults (orange) and older adults (red) groups are plotted. Dashed colored lines indicate the mean group latency from individually scored P300 peak latencies. Scalp topography maps show voltage distributions at mean group peak latencies.

Grand average waveforms with peak topographic maps for both groups are presented in Fig 3. For younger adults, 25 of 26 participants had both a P300 response to the FN and UFN, and for older adults, 13 of 13 participants had both a P300 response to the FN and UFN.

Descriptive statistics for the amplitude and latency for both groups, the three ROIs, and both conditions are presented in Table 2. Both groups exhibited large P300s to both novel sounds, with the familiar novel (FN) generating the larger amplitude on average.

**Table 2:** Means and standard errors of the mean of P300 peak amplitudes and latencies in response to novel stimuli in the oddball mismatch.

ROI	Condition	Younger Adults		Older Adults	
		Amplitude (SEM)	Latency (SEM)	Amplitude (SEM)	Latency (SEM)
Mid Frontal	FN	6.58 (0.43)	364.4 (4.0)	9.49 (0.60)	353.3 (6.2)
	UFN	5.87 (0.51)	362.4 (4.2)	7.71 (0.75)	336.6 (7.1)
Mid Central	FN	8.26 (0.38)	366.6 (3.8)	9.47 (0.56)	345.9 (4.1)
	UFN	6.49 (0.44)	359.2 (4.6)	8.72 (0.64)	330.5 (5.0)
Mid Parietal	FN	9.18 (0.37)	375.7 (4.0)	7.53 (0.56)	377.0 (5.7)
	UFN	6.92 (0.37)	372.2 (5.0)	7.04 (0.61)	365.9 (9.7)

To first determine if there was a significant difference between the two P3 generating conditions and the standard baseline condition, and if there were any group differences, separate mixed-design ANOVAs were conducted for amplitude and latency with *Group* (younger and older adults) as a between-subjects factor, and *Condition* (Standard, Familiar novel, Unfamiliar novel) as a within-subjects factor. There was only a main effect of condition for amplitude (F(2,74) = 55.390, *p* < 0.001), and both a main effect of group (F(1,37) = 7.736, *p* < 0.001) and condition (F(2,74) = 29.031, *p* < 0.001) for latency.

Post-hoc tests showed that all three conditions differed significantly from each other in terms of amplitude (all *p*′ *s* < 0.001), but only the FN and UFN conditions differed compared to the standard condition in terms of latency (all *p*′ *s* < 0.001).

To better understand the difference between the two P3 generating conditions and their scalp distribution, another pair of mixed-design ANOVAs were conducted for amplitude and latency with *Group* (younger and older adults) as a between-subjects factor, and *Condition* (Familiar novel and Unfamiliar novel) and *ROI* (Mid Frontal, Mid Central, and Mid Parietal) as within-subjects factors.

For amplitude, there were main effects of both condition (F(1,37) = 5.020, *p* = 0.031) and ROI (F(2,74) = 3.758, *p* = 0.048), a Group x ROI interaction (F(2,74) = 11.055, *p* < 0.001), and a Group x Condition x ROI interaction (F(2,74) = 10.507, *p* < 0.001).

**Fig 4:**
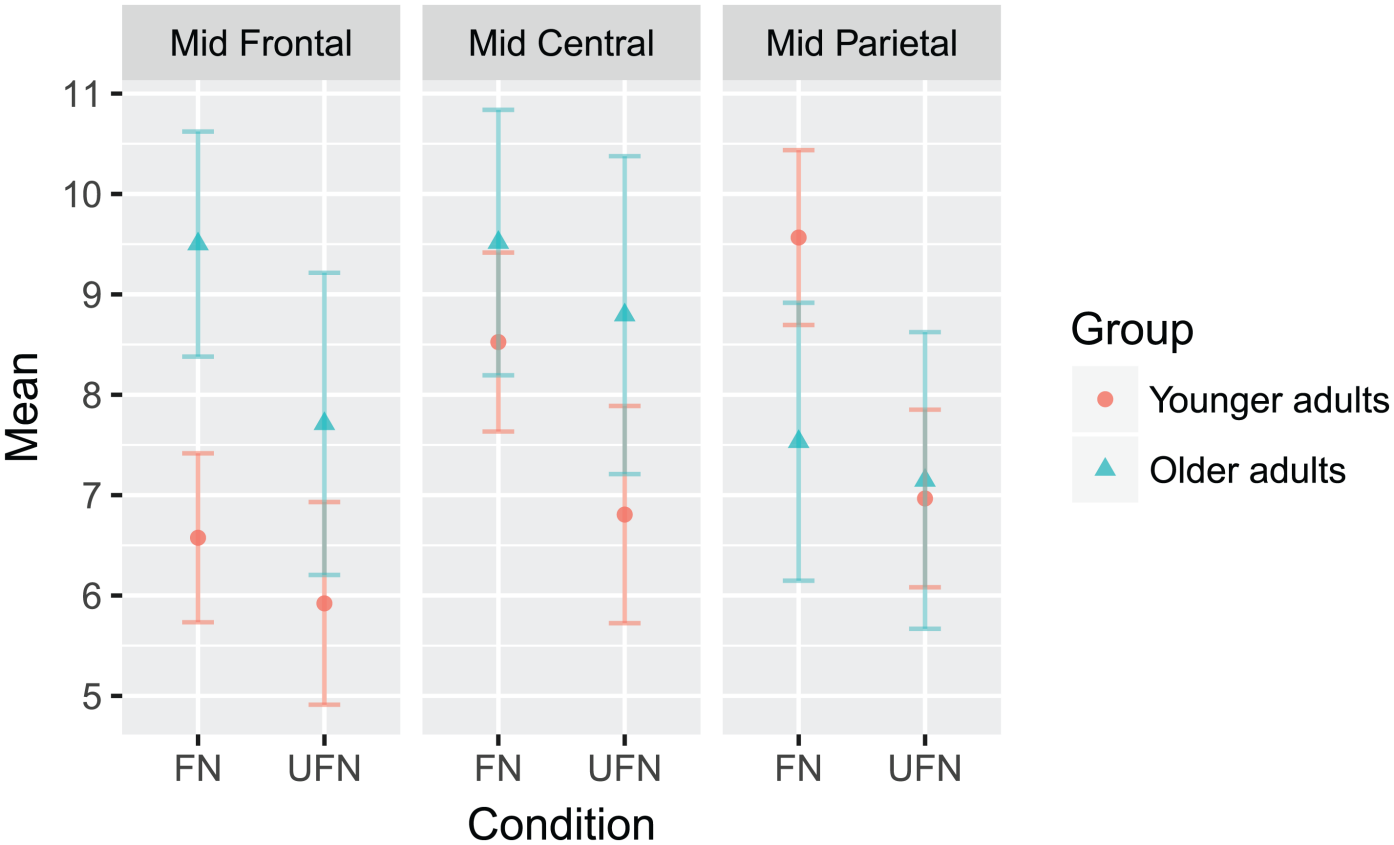
The amplitude Group x Condition x ROI interaction to the P300 response within the oddball mismatch. The mean values of each combination of group, condition, and ROI are plotted. Younger adults are represented with red circles, and older adults with blue triangles. Error bars represent ±0.5 SEM.

Post hoc tests showed that overall, the FN was significantly more positive than the UFN, the Mid Central ROI was significantly more positive than the Mid Frontal ROI, but the Mid Parietal ROI was not significantly different than the other ROIs. The three-way interaction is shown graphically in Fig 4.

For latency, there was only a main effect of ROI (F(2,74) = 11.882, *p* < 0.001), where the P300 scored in the Mid Parietal ROI peaked significantly later than those in the other two ROIs (all *p*′ *s* < 0.001).

### Pattern violation mismatch

**Fig 5:**
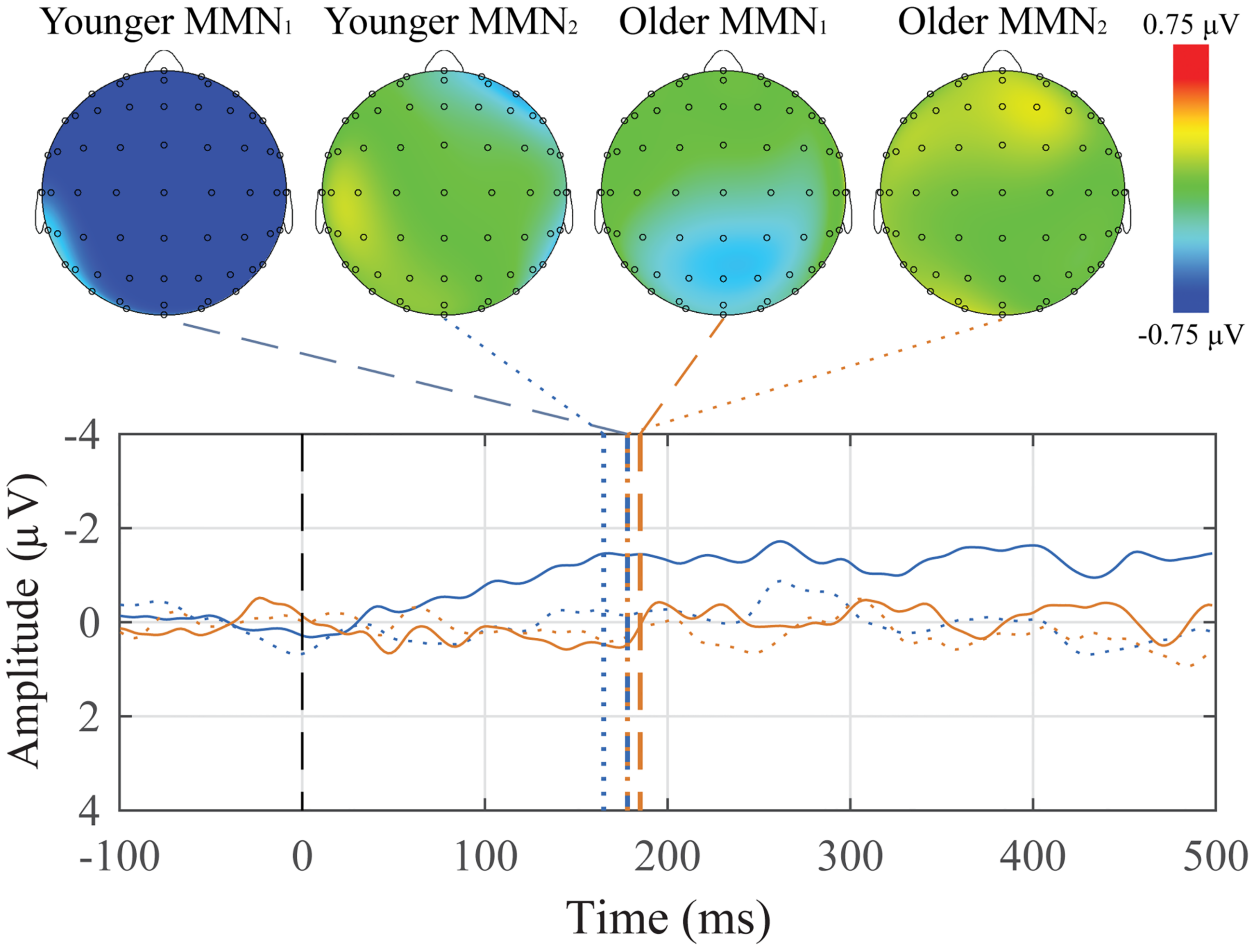
Grand average difference waveforms in the Mid Frontal ROI of the pattern violation mismatch MMN to the first and second deviants and corresponding peak topographic maps. The mean difference response to the first and second deviants for the younger adult and older adult groups are plotted. Young adult first deviant (blue), younger adult second deviant (orange), older adult first deviant (green), and older adult second deviant (red). Dashed colored lines indicate the mean group latency from individually scored MMN peak latencies. Scalp topography maps show voltage distributions at mean group peak latencies.

Grand average waveforms and corresponding peak topographic maps for both groups are presented in Fig 5. For younger adults, 7 of 25 participants (one participant did not complete this paradigm) showed a MMN to the pattern violation mismatch. For older adults, 4 of 12 participants (one participant did not complete this paradigm) showed a MMN to the pattern violation mismatch.

Two difference waves were generated: the difference between the first deviant and the standard (MMN_1_), and the difference between the repetition of the deviant and the standard (MMN_2_).

A summary of the mean amplitudes and latencies of these peaks for each group is presented in Table 3.

**Table 3:** Means and standard errors of the mean of MMN peak amplitudes and latencies for pattern violation mismatches.

ROI	Condition	Younger Adults		Older Adults	
		Amplitude (SEM)	Latency (SEM)	Amplitude (SEM)	Latency (SEM)
Mid Frontal	MMN_1_	-1.69 (0.15)	177.5 (2.6)	-0.17 (0.11)	185.4 (3.4)
	MMN_2_	-0.86 (0.14)	165.3 (2.7)	-0.05 (0.24)	177.5 (4.4)
Mid Central	MMN_1_	-1.54 (0.01)	174.1 (2.2)	-0.40 (0.07)	181.1 (3.1)
	MMN_2_	-0.71 (0.12)	169.7 (2.2)	-0.20 (0.17)	173.1 (3.8)
Mid Parietal	MMN_1_	-1.37 (0.11)	173.2 (2.3)	-0.62 (0.09)	180.0 (3.0)
	MMN_2_	-0.45 (0.12)	175.1 (2.8)	-0.33 (0.12)	172.3 (3.8)

The results from the MMN-detection t-tests are given in Table 4. P-values have been adjusted using a per-group Bonferroni correction.

**Table 4:** Means and standard errors of the mean of MMN peak amplitudes and latencies for pattern violation mismatches.

Group	ROI	Condition	t-value	Degrees of Freedom	Adjusted p-value
Younger Adults	Mid Frontal	MMN_1_	-10	100	<0.001
		MMN_2_	-6	100	<0.001
	Mid Central	MMN_1_	-10	100	<0.001
		MMN_2_	-6	100	<0.001
	Mid Parietal	MMN_1_	-10	100	<0.001
		MMN_2_	-3	100	0.003
Older Adults	Mid Frontal	MMN_1_	-2	50	0.370
		MMN_2_	-0.4	50	1.000
	Mid Central	MMN_1_	-5	70	<0.001
		MMN_2_	-1	70	0.754
	Mid Parietal	MMN_1_	-7	70	<0.001
		MMN_2_	-3	70	0.0277

Since only the response to the first deviant in the mid central and mid parietal ROIs was found to be significantly different from zero, the mixed-design ANOVAs were separately conducted for amplitude and latency with *Group* (younger and older adults) as a between-subjects factor, and *ROI* (Mid Central and Mid Parietal) as a within-subjects factor, and only included peaks from the first deviant.

A significant main effect of Group (F(1,35) = 5.740, *p* = 0.022) and a Group x ROI interaction (F(1,35) = 6.920, *p* = 0.008) were found for peak amplitude. The younger adults had more negative peaks across both regions, with the mid central region being most negative. The older adults, however, had a more negative response in the mid parietal region than compared to the mid central. There were no significant differences with regard to the latencies.

### Subject’s Own Name (SON)

**Fig 6:**
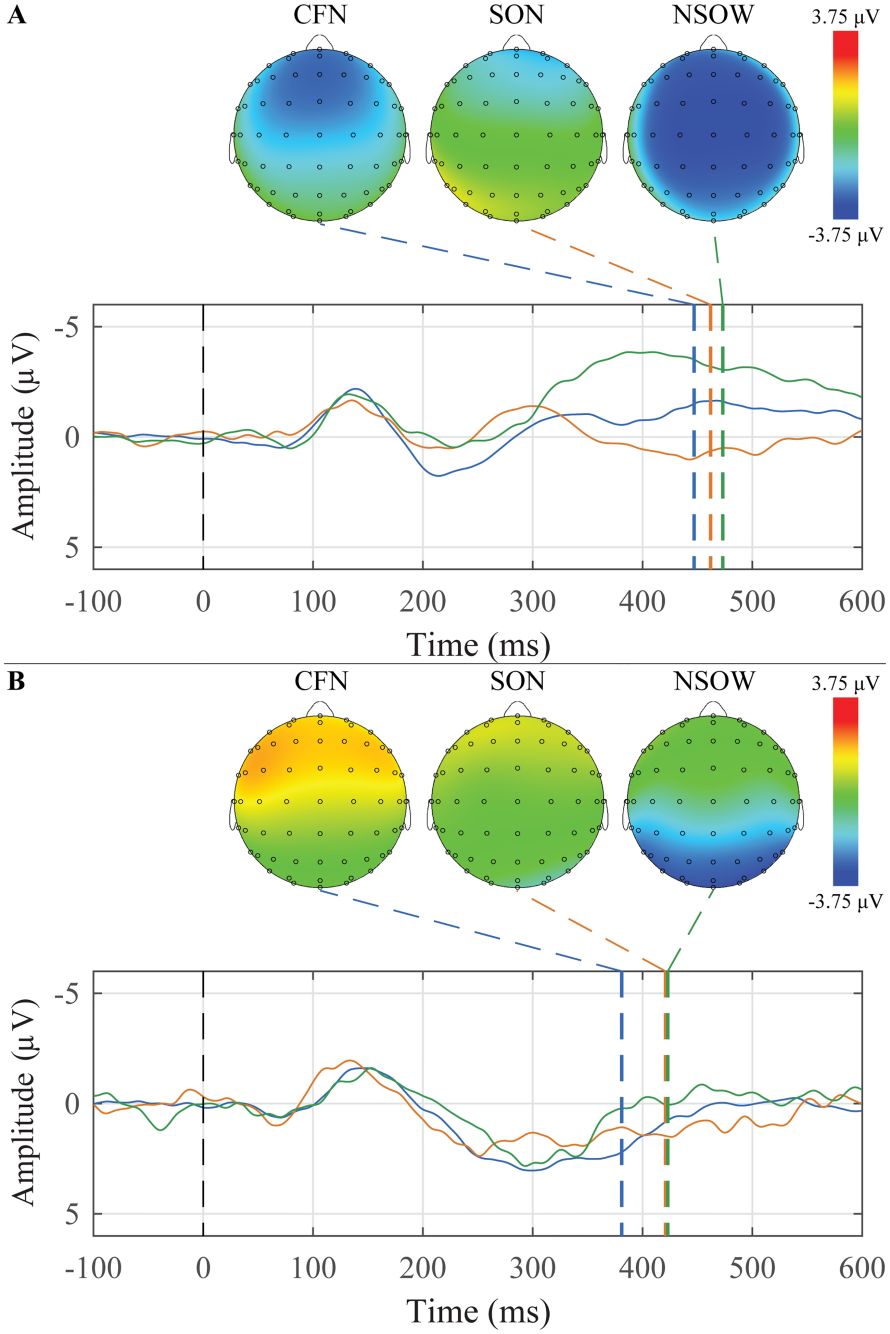
Grand average waveforms to a list of Common First Names, the Subject’s Own Name, and a list of Non-salient Other Words and their corresponding peak topographic maps within the Subject’s Own Name paradigm. A: The younger adult group’s average responses in the Mid Parietal ROI to the Common First Names (blue), Subject’s Own Name (orange), and the list of Non-salient Other Words (green) are plotted. B: The older adult group’s average responses in the Mid Frontal ROI to the Common First Names (blue), Subject’s Own Name (orange), and the list of Non-salient Other Words (green) are plotted.

The grand average waveforms and peak topographic maps for SON, Common First Names, and Non-salient Other Words for younger and older adults are presented in Fig 6. For younger adults, 10 of 26 participants had a P300 response to their own name, and for older adults, 6 of 13 participants had a P300 response to their own name.

Descriptive statistics for the amplitude and latency for both groups, the three ROIs, and both conditions are presented in Table 5.

**Table 5:** Means and standard errors of the mean of P300 peak amplitudes and latencies for the Subjects Own Name paradigm.

ROI	Condition	Younger Adults		Older Adults	
		Amplitude (SEM)	Latency (SEM)	Amplitude (SEM)	Latency (SEM)
Mid Frontal	SON	-0.10 (0.33)	444.8 (7.3)	2.44 (0.34)	420.8 (9.4)
	NSOW	-1.58 (0.37)	451.4 (7.0)	1.90 (0.48)	422.8 (11.8)
	CFN	-1.01 (0.18)	414.1 (7.8)	2.45 (0.33)	381.2 (7.6)
Mid Central	SON	0.54 (0.28)	433.2 (5.4)	1.69 (0.27)	428.1 (8.2)
	NSOW	-1.63 (0.28)	450.6 (6.0)	1.53 (0.30)	412.0 (9.9)
	CFN	-0.68 (0.14)	420.8 (6.2)	2.08 (0.23)	387.3 (7.5)
Mid Parietal	SON	1.35 (0.23)	461.6 (5.5)	0.85 (0.29)	482.5 (8.5)
	NSOW	-1.07 (0.25)	472.8 (6.5)	-0.29 (0.23)	428.3 (10.9)
	CFN	-0.20 (0.12)	446.8 (6.4)	0.89 (0.14)	432.7 (10.6)

Mixed-design ANOVAs were conducted separately for amplitude and latency with *Group* (younger and older adults) as a between-subjects factor, and *Condition* (Subject’s Own Name, Non-salient Other Words, and Common First Names) and *ROI* (Mid Frontal, Mid Central, and Mid Parietal) as within-subjects factors.

For peak amplitude, there were significant main effects of group (F(1,37) = 11.05, *p* = 0.002) and condition (F(2,74) = 4.13, *p* = 0.025). There were also significant Group x ROI (F(2,74) = 31.17, *p* < 0.001) and Condition x ROI (F(4,148) = 3.99, *p* = 0.015) interactions. Generally, the older adults had peaks that were more positive than the younger adults. The Own Name condition was the most positive, followed by other names, and then other words. The interaction effects appear to be driven by the mid parietal region, where the difference between the two groups is smaller.

For peak latency, there was only a significant main effect of ROI (F(2,74) = 18.529, *p* < 0.001) with the mid parietal peaks occurring later than the other two regions.

### Semantic violation sentences

**Fig 7:**
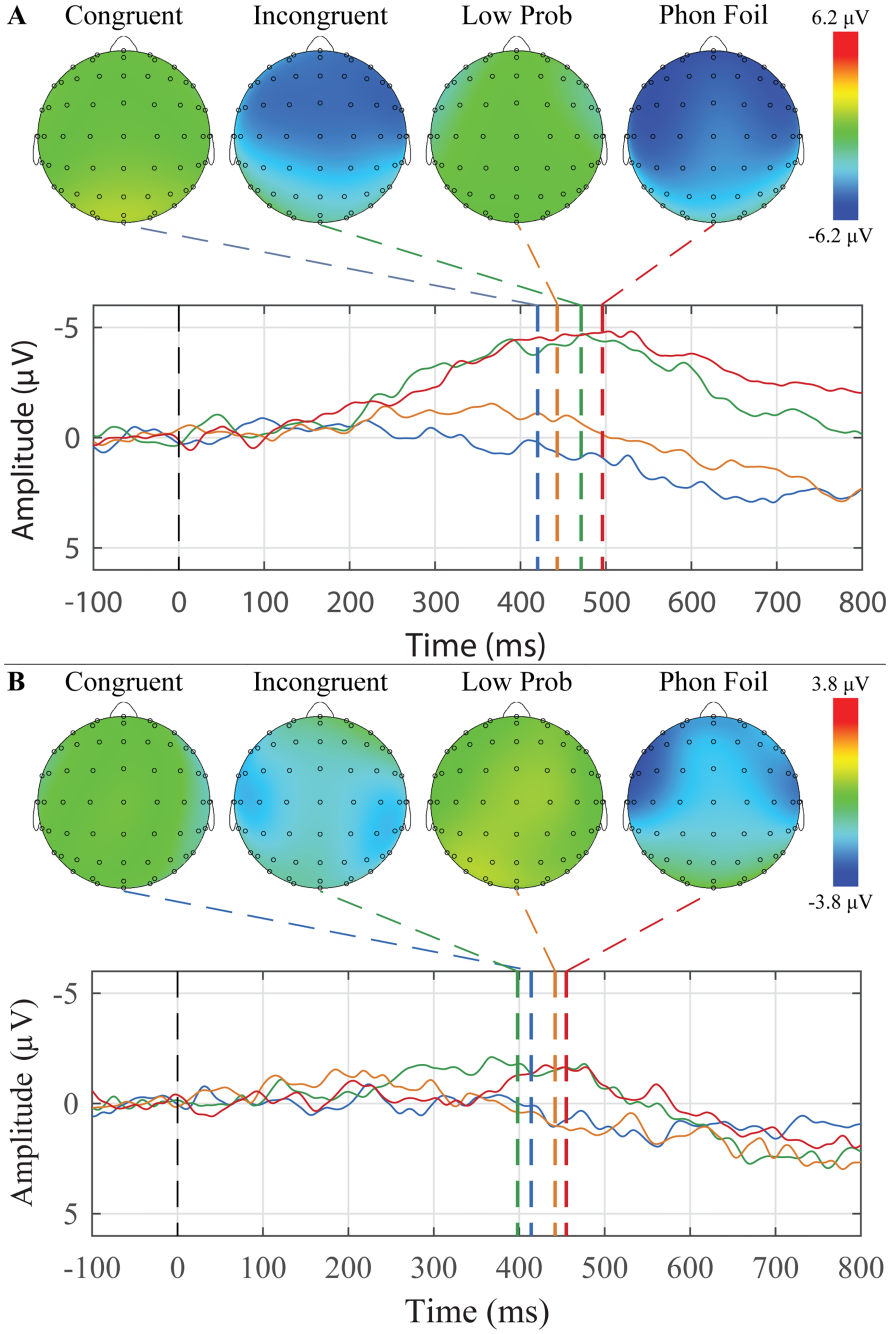
Grand average waveforms to Congruent, Incongruent, Low Probability, and Phonological Foil terminal words and their corresponding peak topographic maps within the semantic violation sentences paradigm. A: The younger adult group’s average responses in the Mid Central ROI to the list of Congruent (blue), Low Probability (orange), Incongruent (green) and Phonological Foil (red) terminal words are plotted. B: The older adult group’s average responses in the Mid Parietal ROI to the list of Congruent (blue), Low Probability (orange), Incongruent (green) and Phonological Foil (red) terminal words are plotted. Dashed colored lines indicate the mean group latency from individually scored N400 peak latencies. Scalp topography maps show voltage distributions at mean group peak latencies.

The grand average waveforms and peak topographic maps for the semantic violation sentences for younger and older adults are presented in Fig 7. N400 peaks were scored for each condition, group, and ROI. The mean amplitudes and latencies are presented in Table 6. For younger adults, 19 of 26 participants had a N400 response to incongruent terminal words, and for older adults, 5 of 13 participants had a N400 response to incongruent terminal words.

**Table 6:** Means and standard errors of the mean of N400 peak amplitudes and latencies for semantic violation sentences.

ROI	Condition	Younger Adults		Older Adults	
		Amplitude (SEM)	Latency (SEM)	Amplitude (SEM)	Latency (SEM)
Mid Frontal	Incongruent	-5.22 (0.29)	493.2 (9.8)	-2.56 (0.36)	471.8 (14.3)
	Low Probability	-2.94 (0.35)	449.9 (12.1)	-1.68 (0.33)	478.2 (19.7)
	Phonological Foil	-5.66 (0.34)	507.7 (10.7)	-2.35 (0.38)	458.4 (15.6)
	Congruent	-1.48 (0.36)	436.9 (10.0)	-1.58 (0.36)	547.1 (17.3)
Mid Central	Incongruent	-5.34 (0.22)	471.3 (7.5)	-2.69 (0.30)	448.4 (10.5)
	Low Probability	-2.77 (0.25)	443.3 (8.8)	-1.61 (0.03)	463.7 (15.3)
	Phonological Foil	-5.47 (0.23)	496.4 (7.8)	-2.64 (0.28)	452.8 (10.1)
	Congruent	-1.59 (0.25)	419.7 (8.6)	-1.12 (0.32)	507.8 (15.1)
Mid Parietal	Incongruent	-4.28 (0.20)	429.9 (6.4)	-2.87 (0.34)	397.8 (8.1)
	Low Probability	-2.31 (0.22)	429.5 (8.1)	-1.38 (0.16)	442.4 (14.5)
	Phonological Foil	-5.15 (0.22)	476.3 (6.4)	-2.07 (0.22)	455.3 (10.1)
	Congruent	-0.91 (0.20)	401.8 (8.5)	-0.80 (0.33)	414.0 (11.3)

Mixed-design ANOVAs were separately conducted for amplitude and latency with *Group* (younger and older adults) as a between-subjects factor, and *Condition* (Congruent, Incongruent, Phonological Foil, and Low Probability) and *ROI* (Mid Frontal, Mid Central, and Mid Parietal) as within-subjects factors. For amplitude, there were significant main effects of Group (F(1,37) = 11.783, *p* = 0.001), Condition (F(3,111) = 12.364, *p* < 0.001), and ROI (F(2,74) = 9.297, *p* = 0.003). Young adult peaks were overall significantly more negative than older adults (*p* < 0.001). Post hoc comparisons showed that Incongruent and Phonological Foil sentences were both significantly more negative than Low Probability sentences (*p*′ *s* < 0.001), which in turn were significantly more negative than Congruent (*p* = 0.007). There was no significant difference between Incongruent and Phonological Foil sentences. The mid parietal ROI was significantly had peaks that were significantly more negative than both the mid frontal and mid central ROIs, but those two ROIs were not significantly different from each other (*p*′ *s* < 0.001).

**Fig 8:**
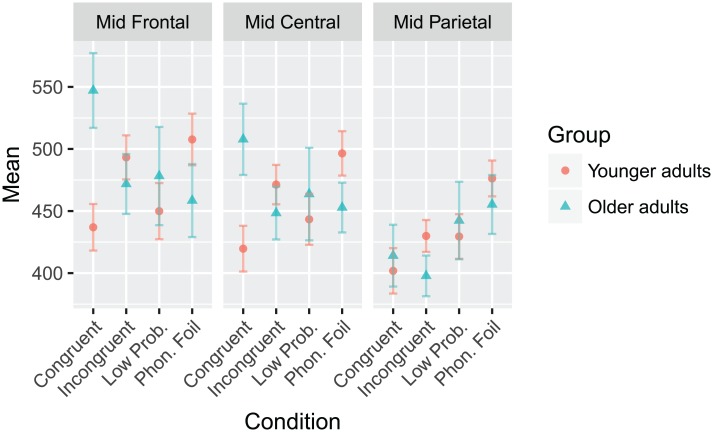
The latency Group x Condition x ROI interaction to the N400 response for the semantic violation sentences paradigm. The mean values of each combination of group, condition, and ROI are plotted. Younger adults are represented with red circles, and older adults with blue triangles. Error bars represent ±0.5 SEM.

For latency, there was a significant main effect of ROI (F(2,74) = 32.457, *p* < 0.001), as well as Group x Condition (F(3,111) = 3.521, *p* = 0.026), Condition x ROI (F(6,222) = 3.990, *p* = 0.003), and Group x Condition x ROI (F(6,222) = 2.898, *p* = 0.019) interactions. Overall, peaks in the mid parietal region occurred earlier than both of the other regions (*p*′ *s* < 0.001), The three-way interaction is shown graphically in Fig 8.

### Word-word priming

**Fig 9:**
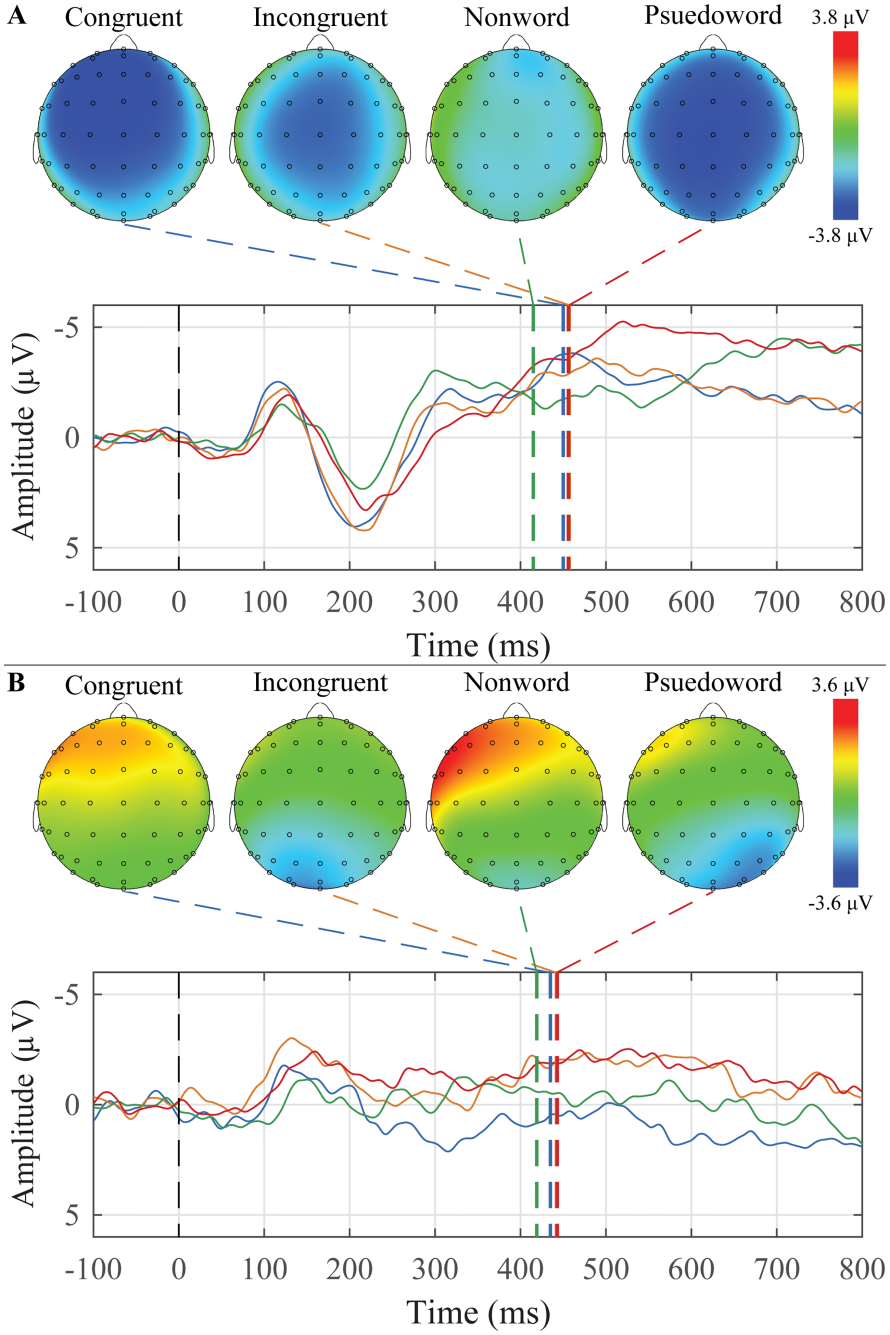
Grand average waveforms to Congruent, Incongruent, Nonword, and Pseudoword target words and their corresponding peak topographic maps within the word-word priming paradigm. A: The younger adult group’s average responses in the Mid Central ROI to the list of Congruent (blue), Incongruent (orange), Nonword (green) and Pseudoword (red) target words are plotted. B: The older adult group’s average responses in the Mid Parietal ROI to the list of Congruent (blue), Incongruent (orange), Nonword (green) and Pseudoword (red) target words are plotted. Dashed colored lines indicate the mean group latency from individually scored N400 peak latencies. Scalp topography maps show voltage distributions at mean group peak latencies.

The grand average waveforms and peak topographic maps for the word-word priming paradigm for younger and older adults are presented in Fig 9. N400 peaks were scored for each condition and each group. The mean amplitudes and latencies are presented in Table 7. For younger adults, 7 of 25 participants (one participant did not complete this paradigm) had a N400 response to incongruent target words, and for older adults, 4 of 11 participants (two participants did not complete this paradigm) had a N400 response to incongruent target words.

**Table 7:** Means and standard errors of the mean of N400 peak amplitudes and latencies for word-word priming.

ROI	Condition	Younger Adults		Older Adults	
		Amplitude (SEM)	Latency (SEM)	Amplitude (SEM)	Latency (SEM)
Mid Frontal	Incongruent	-3.64 (0.41)	462.3 (3.0)	-0.58 (0.48)	451.7 (5.5)
	Pseudoword	-4.36 (0.42)	455.7 (4.3)	0.83 (0.39)	456.5 (5.8)
	Nonword	-3.40 (0.44)	429.7 (5.5)	0.56 (0.57)	397.6 (7.6)
	Congruent	-3.86 (0.38)	444.4 (4.3)	1.30 (0.57)	461.2 (5.6)
Mid Central	Incongruent	-4.06 (0.35)	457.1 (2.7)	-1.49 (0.40)	451.3 (4.7)
	Pseudoword	-4.70 (0.29)	455.8 (3.6)	-0.74 (0.33)	449.6 (5.6)
	Nonword	-3.14 (0.35)	415.0 (4.4)	0.50 (0.34)	400.5 (6.2)
	Congruent	-3.72 (0.32)	450.0 (3.2)	0.95 (0.47)	456.4 (5.2)
Mid Parietal	Incongruent	-3.58 (0.30)	451.7 (3.3)	-2.73 (0.42)	441.9 (5.5)
	Pseudoword	-4.20 (0.21)	451.7 (3.5)	-2.62 (0.28)	442.7 (5.9)
	Nonword	-3.26 (0.30)	405.4 (3.7)	-0.47 (0.24)	418.8 (6.3)
	Congruent	-3.29 (0.28)	436.5 (3.9)	-0.40 (0.39)	435.3 (6.8)

Mixed-design ANOVAs were conducted separately for amplitude and latency with *Group* (younger and older adults) as a between-subjects factor, and *Condition* (Semantically Congruent, Semantically Incongruent, Non-word, and Pseudoword) and *ROI* (Mid Frontal, Mid Central, and Mid Parietal) as within-subjects factors.

For amplitude, there were significant main effects of group (F(1,34) = 10.424, *p* = 0.003) and ROI (F(2,68) = 8.499, *p* = 0.003), and a Group x ROI interaction (F(2,68) = 23.155, *p* < 0.001). Younger adults peaks were generally more negative than the older adults, and the mid parietal region was significantly more negative than the mid frontal region overall. Older adults appear to have a more focused negativity towards the mid parietal region, whereas the younger adults have a large negativity that is present across all three regions.

**Fig 10:**
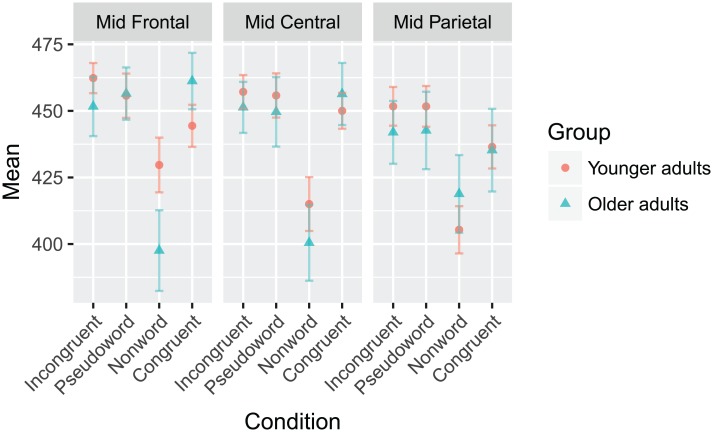
The latency Group x Condition x ROI interaction to the N400 response for the word-word priming paradigm. The mean values of each combination of group, condition, and ROI are plotted. Younger adults are represented with red circles, and older adults with blue triangles. Error bars represent ±0.5 SEM.

There were main effects of condition (F(3,102) = 8.488, *p* < 0.001) and ROI (F(2,68) = 6.015, *p* = 0.009) in terms of peak latency, as well as a significant Group x Condition x ROI interaction (F(6,204) = 3.765, *p* = 0.009). All non-word targets had significantly earlier peaks compared to all other conditions (all *p*’s < 0.001), and peaks in the mid parietal region occurred significantly earlier than those in the other two regions (all *p*’s < 0.001). The three-way interaction is shown graphically in Fig 10.

### Behavioral manipulation

The analysis for the three paradigms used in the behavioral manipulation was the same as described above.

#### SON

**Fig 11:**
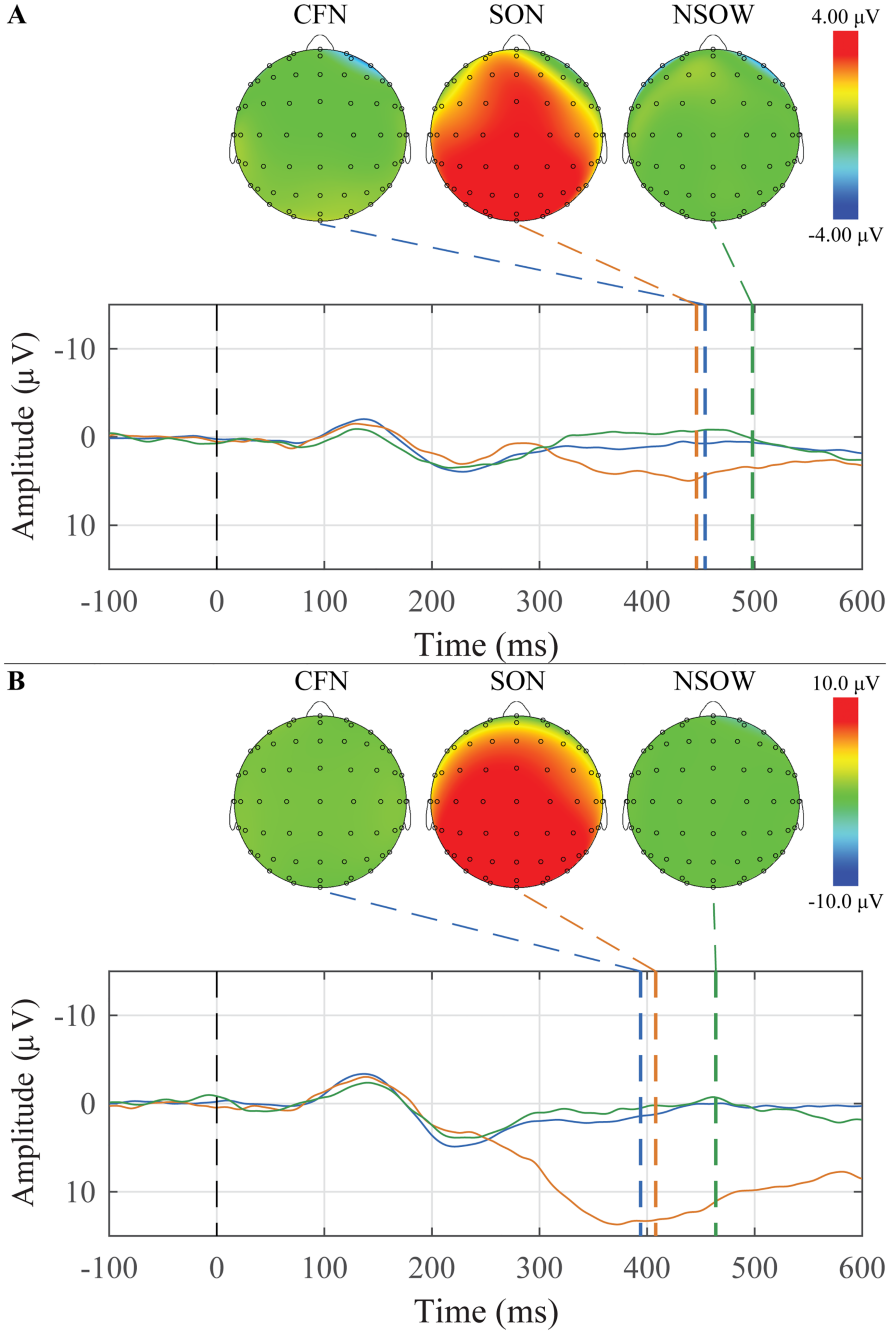
Grand average waveforms at Cz to a list of Common First Names, the Subject’s Own Name, and a list of Non-salient Other Words and their corresponding peak topographic maps within the Subject’s Own Name paradigm with the behavioral manipulation. A: The average responses to the Common First Names (blue), Subject’s Own Name (orange), and the list of Non-salient Other Words (green) in the Passive condition are plotted. B: The average responses to the Common First Names (blue), Subject’s Own Name (orange), and the list of Non-salient Other Words (green) in the Active condition are plotted. Dashed colored lines indicate the mean group latency from individually scored P300 peak latencies. Scalp topography maps show voltage distributions at mean group peak latencies.

The grand average waveforms and peak topographic maps for SON, Common First Names, and Non-salient Other Words for the Passive and Active tasks are presented in Fig 11. Descriptive statistics for the amplitude and latency for both groups, the three ROIs, and both conditions are presented in Table 8. In the passive condition, 9 of 13 participants had a P300 response to their own name, whereas in the active condition, 13 of 13 participants had a P300 response to their own name.

**Table 8:** Means and standard errors of the mean of P300 peak amplitudes and latencies for the active and passive versions of the Subjects Own Name paradigm.

ROI	Condition	Active		Passive	
		Amplitude (SEM)	Latency (SEM)	Amplitude (SEM)	Latency (SEM)
Mid Frontal	SON	9.86 (1.00)	385.1 (5.7)	4.14 (0.44)	418.1 (9.8)
	NSOW	2.52 (0.65)	411.5 (10.8)	3.17 (0.96)	452.3 (13.1)
	CFN	2.79 (0.29)	385.7 (9.6)	1.82 (0.80)	454.3 (14.5)
Mid Central	SON	13.28 (0.81)	400.5 (7.2)	4.92 (0.50)	425.6 (7.6)
	NSOW	2.58 (0.45)	444.3 (10.7)	2.94 (0.91)	477.3 (10.5)
	CFN	2.77 (0.23)	387.8 (7.2)	2.2 (0.70)	449.5 (11.1)
Mid Parietal	SON	15.46 (0.84)	408.1 (7.2)	5.46 (0.53)	446.2 (7.7)
	NSOW	2.69 (0.41)	464.4 (11.4)	3.05 (0.96)	498.3 (10.8)
	CFN	2.38 (0.2)	394.0 (6.9)	2.45 (0.73)	454.0 (9.8)

**Fig 12:**
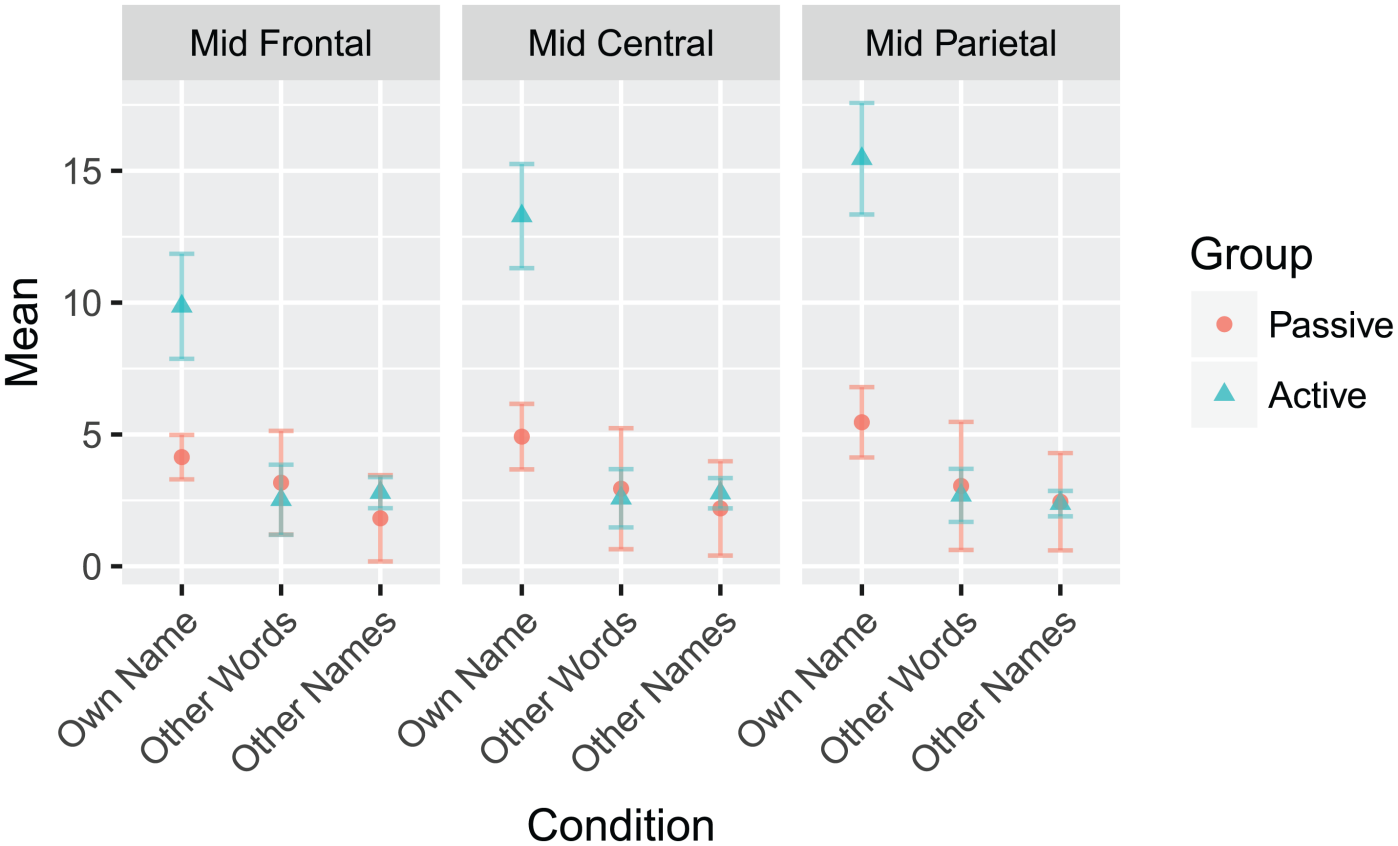
The amplitude Behavioural Condition x Task Condition x ROI interaction to the P3 response for the Subject’s Own Name paradigm. The mean values of each combination of behavioural condition, task condition, and ROI are plotted. The passive task condition is represented with red circles, and the active task condition with blue triangles. Error bars represent ±0.5 SEM.

Repeated measures ANOVAs were conducted separately for amplitude and latency with *Behavioral Condition* (Passive and Active), *Task Condition* (Subject’s Own Name, Non-salient Other Words, and Common First Names), and *ROI* (Mid Frontal, Mid Central, and Mid Parietal) as factors. For amplitude, there were significant main effects of task condition (F(2,44) = 20.370, *p* < 0.001) and ROI (F(2,44) = 5.760, *p* = 0.019), as well as Behavioural Condition x Task Condition (F(2,44) = 8.450, *p* = 0.004), Task Condition x ROI (F(4,88) = 11.210, *p* < 0.001), and Behavioural Condition x Task Condition x ROI (F(4,88) = 5.530, *p* = 0.009) interactions. Peaks to the subject’s own name condition were significantly more positive than the other two conditions across behavioural condition and regions. Peaks were also, on average, significantly more positive in the mid parietal region than in the mid frontal region, but no significant difference was found between the mid central region and the other regions. The Behaviour x Task Condition interaction is attributable to the larger P300 amplitude to the SON in the Active compared to the Passive condition; an effect not found in the other two task conditions. The three-way interaction is shown graphically in Fig 12.

Peak latency had only significant main effects of behavioural condition (F(1,22) = 5.465, *p* = 0.029), task condition (F(2,44) = 4.190, *p* = 0.030), and ROI (F(2,44) = 4.190, *p* = 0.008). Overall, the P3 peaked earlier in the active condition compared to the passive condition. The P3 also peaked earlier to the subject’s own name and to other names than to other words, and was on average later in the mid parietal region than the other two regions.

#### Semantic violation sentences

**Fig 13:**
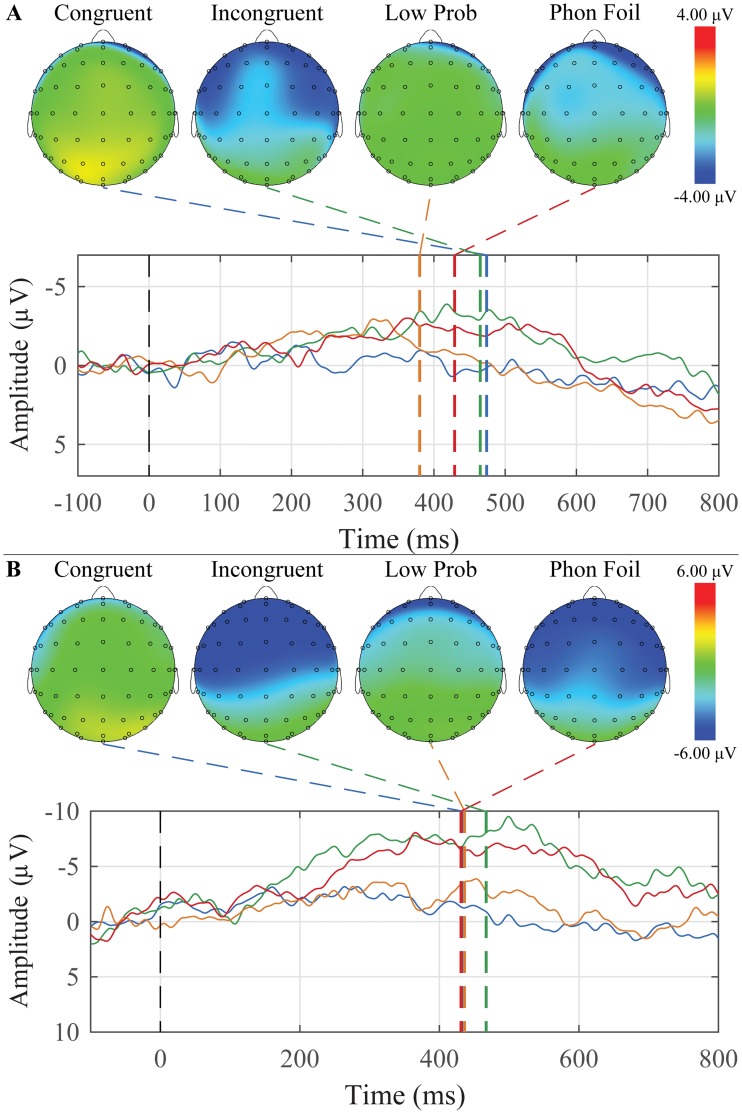
Grand average waveforms at Cz to Congruent, Incongruent, Low Probability, and Phonological Foil terminal words and their corresponding peak topographic maps within the semantic violation sentences paradigm with the behavioral manipulation. A: The average responses to the list of Congruent (blue), Low Probability (orange), Incongruent (green) and Phonological Foil (red) terminal words in the Passive condition are plotted. B: The average responses to the list of Congruent (blue), Low Probability (orange), Incongruent (green) and Phonological Foil (red) terminal words in the Active condition are plotted. Dashed colored lines indicate the mean group latency from individually scored N400 peak latencies. Scalp topography maps show voltage distributions at mean group peak latencies.

The grand average waveforms and peak topographic maps for the semantic violation sentences for the Passive and Active tasks are presented in Fig 13. N400 peaks were scored for each task condition and each behavioral condition. The mean amplitudes and latencies are presented in Table 9. In the passive condition, 4 of 13 participants had a N400 response to incongruent terminal words, whereas in the active condition, 9 of 13 participants had a N400 response to incongruent terminal words.

**Table 9:** Means and standard errors of the mean of N400 peak amplitudes and latencies for the active and passive versions of semantic violation sentences.

ROI	Condition	Active		Passive	
		Amplitude (SEM)	Latency (SEM)	Amplitude (SEM)	Latency (SEM)
Mid Frontal	Incongruent	-10.29 (0.84)	467.3 (13.4)	-5.26 (1.03)	465.2 (17.8)
	Low Probability	-4.98 (0.94)	435.8 (14.4)	-3.34 (0.65)	379.6 (13.7)
	Phonological Foil	-10.16 (1.10)	431.4 (13.8)	-4.42 (0.60)	429.1 (14.6)
	Congruent	-5.03 (0.87)	432.6 (18.1)	-3.17 (0.59)	473.8 (19.0)
Mid Central	Incongruent	-7.83 (0.45)	427.1 (11.7)	-4.54 (0.80)	453.1 (12.8)
	Low Probability	-3.16 (0.54)	406.1 (11.1)	-2.54 (0.53)	391.1 (11.4)
	Phonological Foil	-7.58 (0.61)	424.4 (8.5)	-4.24 (0.51)	424.2 (11.3)
	Congruent	-3.11 (0.43)	381.9 (11.1)	-2.21 (0.43)	467.3 (15.9)
Mid Parietal	Incongruent	-4.65 (0.31)	407.1 (10.8)	-3.44 (0.81)	417.5 (11.4)
	Low Probability	-1.30 (0.47)	396.1 (11.6)	-1.81 (0.56)	401.3 (10.0)
	Phonological Foil	-4.85 (0.53)	419.3 (8.1)	-3.13 (0.50)	421.8 (9.9)
	Congruent	-1.76 (0.41)	356.5 (7.9)	-1.31 (0.50)	442.7 (14.9)

Repeated measures ANOVAs were conducted separately for amplitude and latency with *Behavioral Condition* (Passive and Active), *Task Condition* (Congruent, Incongruent, Phonological Foil, and Low Probability), and *ROI* (Mid Frontal, Mid Central, and Mid Parietal) as factors. For amplitude, there were significant main effects of Task Condition (F(3,66) = 4.099, *p* = 0.023), and ROI (F(2,44) = 25.535, *p* < 0.001), and a significant Behavioural Condition x ROI interaction (F(2,44) = 5.559, *p* = 0.023). Overall, the response to Incongruent endings was the most negative, although there was no significant difference between Incongruent and Phonological Foil endings. All three regions were significantly different from each other, with the mid frontal being the most negative, and the mid parietal being the least negative. The interaction appears to be driven by a reduction in the difference between the active and passive behavioural condition in the mid parietal region, compared to the other regions.

For latency, there was only a significant main effect of ROI (F(2,44) = 12.036, *p* < 0.001) with mid parietal peaks occurring significantly earlier than those in the mid frontal region.

#### Word-word priming

**Fig 14:**
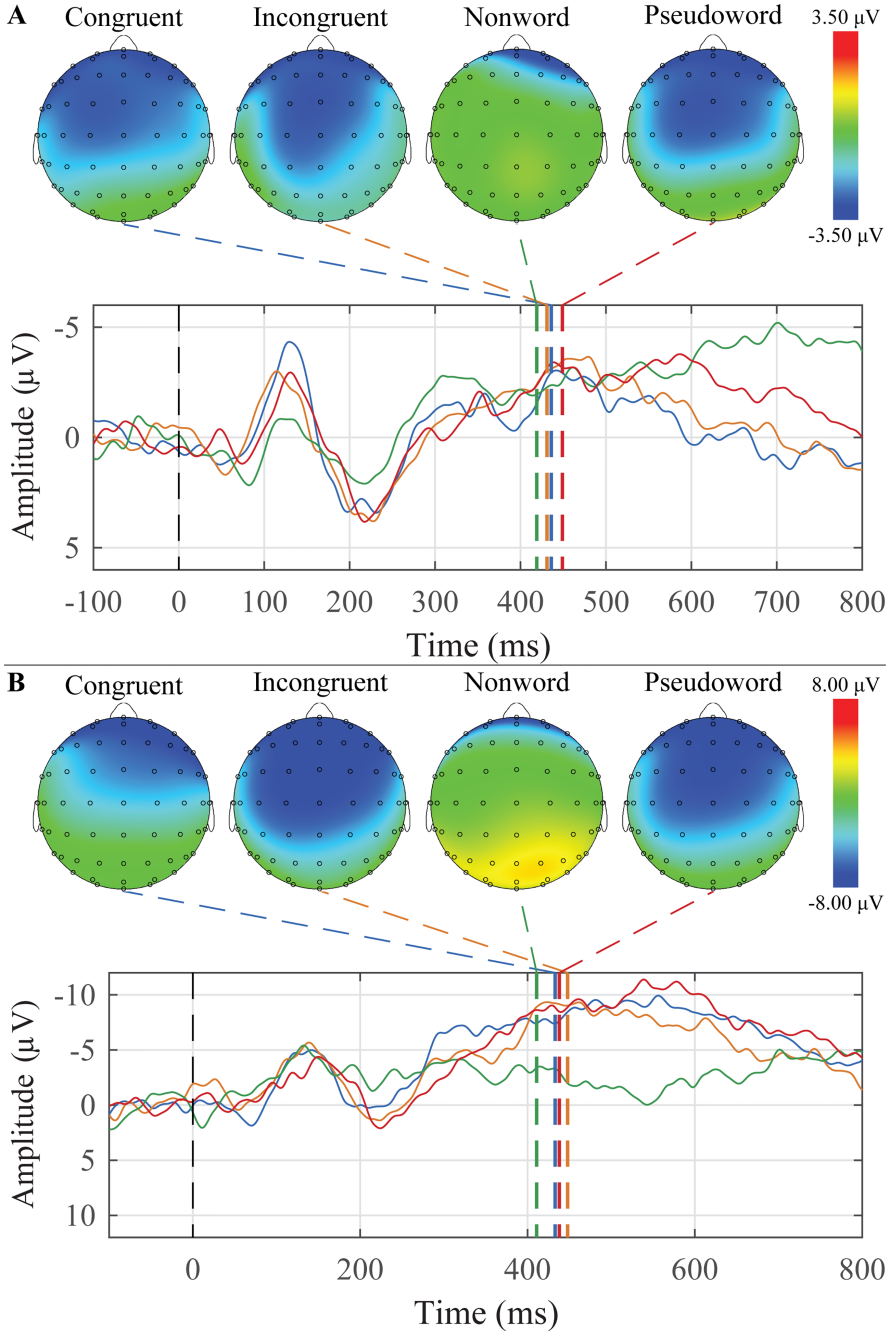
Grand average waveforms at Cz to Congruent, Incongruent, Nonword, and Pseudoword target words and their corresponding peak topographic maps within the word-word priming paradigm with the behavioral manipulation. A: The average responses to the list of Congruent (blue), Incongruent (orange), Nonword (green) and Pseudoword (red) target words in the Passive condition are plotted. B: The average responses to the list of Congruent (blue), Incongruent (orange), Nonword (green) and Pseudoword (red) target words in the Active condition are plotted. Dashed colored lines indicate the mean group latency from individually scored N400 peak latencies. Scalp topography maps show voltage distributions at mean group peak latencies.

The grand average waveforms and peak topographic maps for the word-word priming paradigm for the Passive and Active conditions are presented in Fig 14. N400 peaks were scored for each condition and each group. The mean amplitudes and latencies are presented in Table 10. In the passive condition, 2 of 13 participants had a N400 response to incongruent target words, whereas in the active condition, 5 of 13 participants had a N400 response to incongruent terminal words.

**Table 10:** Means and standard errors of the mean of N400 peak amplitudes and latencies for the active and passive versions of word-word priming.

ROI	Condition	Active		Passive	
		Amplitude (SEM)	Latency (SEM)	Amplitude (SEM)	Latency (SEM)
Mid Frontal	Incongruent	-9.94 (0.65)	448.0 (5.4)	-3.97 (0.73)	430.8 (6.3)
	Pseudoword	-10.29 (1.18)	438.3 (6.7)	-4.06 (0.88)	448.7 (5.4)
	Nonword	-5.10 (1.31)	410.6 (6.6)	-4.22 (0.83)	418.5 (8.0)
	Congruent	-10.46 (1.35)	433.4 (7.0)	-3.80 (0.84)	436.4 (6.6)
Mid Central	Incongruent	-8.84 (0.49)	437.3 (4.0)	-3.77 (0.51)	431.6 (4.8)
	Pseudoword	-8.46 (0.92)	423.3 (5.2)	-3.55 (0.59)	429.1 (4.6)
	Nonword	-1.28 (0.83)	389.3 (4.2)	-2.67 (0.76)	402.1 (6.4)
	Congruent	-7.95 (0.94)	421.5 (6.2)	-3.54 (0.58)	432.1 (6.6)
Mid Parietal	Incongruent	-5.29 (0.46)	434.3 (4.1)	-2.89 (0.39)	442.0 (5.0)
	Pseudoword	-4.71 (0.83)	423.7 (4.2)	-1.80 (0.63)	418.5 (4.6)
	Nonword	1.62 (0.60)	373.0 (3.5)	-1.76 (0.72)	393.1 (6.2)
	Congruent	-3.49 (0.73)	406.6 (6.3)	-2.25 (0.54)	427.5 (6.7)

Repeated measures ANOVAs were conducted separately for amplitude and latency with *Behavioral Condition* (Passive and Active), *Task Condition* (Semantically Congruent, Semantically Incongruent, Non-word, and Pseudoword), and *ROI* (Mid Frontal, Mid Central, and Mid Parietal) as factors.

For peak amplitude, there was a significant main effect of Task Condition (F(3,66) = 4.032, *p* = 0.015) with the responses to the Congruent, Incongruent, and Pseudoword targets all being significantly more negative than the response to Non-word targets (all *p*’s < 0.001), and a significant main effect of ROI (F(2,44) = 26.671, *p* < 0.001), with the mid frontal and mid central regions being significantly more negative than the mid parietal. Additionally, there was a significant Behavioural Condition x Task Condition (F(3,66) = 3.054, *p* = 0.042) interaction where all target words except for the Non-word targets had a more negative response when actively responded to, and a significant Behavioural Condition x ROI (F(2,44) = 7.430, *p* = 0.010) interaction where the active condition became less negative in more posterior sites, but the passive condition was the same throughout.

For peak latency, there were only significant main effects of Task Condition (F(3,66) = 4.841, *p* = 0.005), with Non-word targets occurring significantly earlier than the responses to all other targets (all *p*’s < 0.001), and ROI (F(2,44) = 13.364 *p* < 0.001) with mid parietal peaks occurring earlier than those in the mid frontal region.

## Discussion

We recorded ERPs from 26 younger and 13 older healthy adults in five paradigms eliciting the MMN, P300, and N400 components. As one of the goals of this study was to evaluate and select paradigms that were capable of strongly eliciting the ERP components of interest, we will examine each grouping in turn. The number of participants who exhibited an ERP component in each paradigm is given in Table 11.

**Table 11:** Counts of participants exhibiting the desired ERP response in each paradigm.

**Passive-only**	**Younger**	**Older**
Oddball mismatch (MMN)	21/26	12/13
Pattern violation mismatch	7/25	4/12
Oddball mismatch (P300 to novels)	25/26	13/13
SON	10/26	6/13
Semantic violation sentences	19/26	5/13
Word-word priming	7/25	4/11
**Behavioural manipulation**	**Passive**	**Active**
SON	9/13	13/13
Semantic violation sentences	4/13	9/13
Word-word priming	2/13	5/13

Two paradigms were used to elicit the MMN, differing primarily in the type of violation. As illustrated in Figs 2 and 5, although both paradigms produced negativities, those generated using the classical oddball paradigm (Fig 2) were larger (in some cases 4 μV), and more clearly defined than those seen in the pattern violation paradigm (Fig 5).

The oddball MMN had significant latency differences between the age groups, with older adult participants having later peak latencies compared to the younger adult group, and latency differences between conditions. The pattern violation paradigm did not generate strong MMNs, however there was a difference in amplitudes between groups, with younger participants having amplitudes that were almost four times larger than the older participants. This age-related attenuation effect is consistent with other studies which also found reduced MMN amplitudes at relatively short inter-stimulus intervals in older adult participants. [32–35] Since the strongest and most reliable MMN was generated with the auditory oddball paradigm, that would be one of the paradigms included in the suggested battery.

Two paradigms were used to elicit the P300: novel sounds within an auditory oddball paradigm (Fig 3), and a subject’s own name (SON) paradigm that included other names and words (Fig 6). The difference in the size of the waveforms between the two paradigms is quite noticeable. The N1 and P2 components in the oddball paradigm are almost 300% larger than those in the SON paradigm. Unexpectedly, the P300 to the subject’s own name, which should be quite sizable, is late and very small in the SON paradigm employed here; but appears in the correct time window and is larger when embedded within tones. Within the oddball paradigm, there is a significant difference in amplitude between the familiar and unfamiliar novel conditions and the standard tone. There was also a significant difference between groups and between conditions.

The P300 in the SON paradigm was expected to be largest to the subject’s own name, however that is not the case for the older participants (Fig 6). These participants exhibited a positivity to the list of other common names, but not their own. Younger participants showed a later and sustained positivity to their own name compared to the other common names; even this response is, however, relatively small. Both groups exhibit an N400-like component to the list of other words. Overall, the younger participants displayed larger waveforms, which is seen as a significant main effect of group. These data would appear to support the previous claims that the P300 may not always be a reliable enough measure to be used in a clinical setting. [36–38] As the P300 was most reliably elicited to the novel stimuli in the auditory oddball paradigm without requiring attending to the sounds, we would again recommend the use of this paradigm in the suggested battery. This provides the added benefit of eliciting two components with only one stimulation paradigm.

The N400 was elicited using two paradigms of increasing semantic context: sentences with a terminal-word manipulation (Fig 7) and word-word priming (Fig 9). In both paradigms, no explicit instruction was given to participants to attend to any semantic relations between the words. In the case of the word-word priming, there was again an effect of age with the younger participants having larger N400’s than the older. Across groups, there was a significant difference between the non-word targets and all other target words, with the non-words having earlier peak latencies.

The semantic violation sentences showed significant age effects, with younger participants having larger N400’s than older. They also showed significant condition effects, with incongruent and phonological foil endings resulting in larger N400 amplitudes than the low probability and congruent conditions. The responses to the incongruent endings in the semantic violation sentences were generally more negative than the responses to the incongruent targets in the word-word priming. Considering the results up to this point, the semantic violation sentences appear to have a better ability to elicit the N400 without explicit instruction to attend to the sentences.

To better understand the effect of attention on these paradigms, we recorded ERPs from 13 other young healthy adults while they first passively experienced the stimuli, and then actively responded to what they were hearing.

Overall, the P300 responses to the SON and the N400 responses to semantically incongruent sentence endings and target words were much larger in the active task condition than in the passive task condition. This is in line with the amplitude differences reported in [39] where actively responded to oddball stimuli elicited more positive P300 responses than those that were passively listened to. This gives reason to always provide instruction to attend to the stimuli irrespective of the participant’s ability.

The use of ERPs to assess the clinical state of an individual is not without its complexities. ERPs are in some ways ideally suited to examine the cognitive consequences of brain injury because different ERP components are so strongly related to specific cognitive functions [40].

EEG and ERPs in clinical contexts have many advantages: 1. The ability of most people to tolerate the less intimidating environment that characterize other brain recording systems (e.g., MRI); 2. The close relationship between particular ERP components and particular sensory, perceptual and cognitive processes—a feature shared only with the more expensive magnetoencephalography (MEG) methodology; 3. The lowest costs of any neuroimaging/recording method; and, 4. The exquisite sensitivity of ERP measures to many of the most common manifestations of CNS pathology, particularly acquired brain injury (ABI)–that is, generalized response latency delays, reduced response amplitudes and most notably domain-specific changes in latency/amplitude that reveal compromised functional integrity in attention, memory and language (see [41]).

Over the years, however, there has been the belief that ERPs, and in particular the oddball P300, are not sufficiently stable to serve as clinical tools. [36–38] This view has often been conflated to include later-occurring responses that are related to higher level cognitive functions. While the MMN has long demonstrated its stability and relevance as a clinical tool, there remains some skepticism regarding the oddball P300 [42]. However, when evaluated against gold standard medical assays, the P300 recorded using the oddball paradigm fares well.

Using the coefficient of variation (CV) [43] and normative data from various standard biomedical test norms [44], a comparison was made of these tests’ CVs with CVs derived from literature-based data for P300 amplitude and latency [42]. Lower CVs imply a leptokurtic distribution rather than a platykurtic distribution making lower CVs preferable because any observed atypical response can more reliably be attributed to true abnormality rather than an uncontrolled source of variance. P300 amplitudes’ CV values were comparable to assays for triglycerides used for assessing heart health—these values being amongst the highest in the collection of standard clinical assays evaluated. In contrast, CVs for P300 latencies were comparable to the lowest CVs recorded for standard clinical assays such as those for hemoglobin and potassium, some tests for thyrotropin, and considerably lower than assays for cholesterol and glucose [42].

As impressive as these findings are, however, there remains a question about the test-retest reliability of the oddball P300 in individual subjects; and without reliability at the individual subject level there will be limited adoption of this protocol in clinical settings. Although a strong case is made for the clinical utility of the P300, Polich and Herbst acknowledge that protocols enabling improved sensitivity and discriminability are needed before P300 is adopted more widely for use in clinical settings [42].

Further criticisms of the utility of the P300 in clinical settings include Picton [36], who suggested that it reflected little beyond the fact that an individual was capable of responding differentially to frequently and infrequently occurring stimuli. He also identified the lack of relevance of the oddball P300 for a patient because it measured such a relatively inconsequential activity. A further limitation of the oddball P300 is its non-specificity; the response is frequently found to be delayed in latency and/or smaller in amplitude in a wide range of CNS pathologies but specific to none. These characteristics are not in and of themselves “fatal flaws” for the P300 if the paradigm can be constructed to target a specific function in a highly reliable manner.

[45] used a local/global paradigm and found in several experiments that a late positivity (the P3b) was obtained reliably if and only if participants were consciously aware of the global regularity pattern and violations of that pattern. That is, an unengaged participant did not exhibit a P3b–a finding that is compatible with earlier criticisms of the traditional P300 paradigms. In fact, Bekinschtein et al. included a “mind wandering” condition in their work and demonstrated an absence of the late positivity indicative of a failure to recognize the global stimulus structure and its violations. At the same time, however, the MMN was observed reliably and in accord with their findings in MCS patients was interpreted as reflecting “conscious processing of local regularities”.

Despite concerns surrounding the use of classic oddball paradigms as clinical tests, they retain clinical assessment potential. However, as is often the case with many clinically useful assessment tests, classic oddball tests are best employed in conjunction with other tests that address more specific cognitive processes. Also, the entire test context should not be ignored any more than it would be ignored in a more traditional neuropsychological assessment context. That is, the choice of test, test sequence, and the initial difficulty level should be chosen on the basis of patient performance, and to the extent possible, clinical judgment.

Connolly and colleagues have proposed a complementary set of criticisms and suggestions that address the relevance of the testing paradigms to the individual patient and to the pathology being targeted; an approach that increases the reliability of the P300 at the individual subject level [37, 38]. Building on earlier arguments [36], they have noted that it is possible to address both the relevance to the patient issue and the reliability of the P300 by implementing protocols that are transparently relevant to the patient and ask more of the patient than differentiating stimuli that occur often from those that do not. For example, Connolly and colleagues [46] adapted the vocabulary tests in the Wechsler Intelligence Scale for Children III (WISC-III) [47] and the Wechsler Adult Intelligence Scale-Revised as a Neuropsychological Instrument (WAIS-R-NI) [48] in order to test receptive language skills and vocabulary knowledge in a group of younger adults.

The specificity of the P300 component as an assessment measure emphasizes the importance of task choice. In particular, specificity for the P300 was found to be 90.5% for the WISC-III, that indicated that 19/21 healthy participants showed the expected response statistically while specificity for the WAIS-R was 85.7% (18/21 participants). It is important to note that when these two psychometric tests were combined, the P300 specificity value was 90.5% (19/21 participants).

These specificity values were obtained when participants’ behavior (button presses to correct and incorrect choices) was taken into account by creating the averaged P300 with correctly identified deviant stimuli only. Connolly and colleagues recognized that relying on an ERP component whose specificity was possibly dependent on identifying a participant’s behavioral accuracy and removing data obtained only when participants responded inaccurately might be of little value in trying to assess the cognitive ability of a patient incapable of executing any type of behavioral response. However, when behavioral responses were not taken into account and averaged P300 responses were obtained without removing incorrectly identified trials (thus “simulating” a non-responsive participant), specificity values for the WISC-III actually increased to 95.2% (20/21 participants), but declined for the WAIS-R, 66.6% (14/21 participants). But when the two tests were combined for assessment purposes, specificity was 90.5% (19/21 participants)–precisely the same value obtained when behavior was accounted for. These findings demonstrate not merely the utility of this particular combination of tests in providing significant information about individuals’ cognitive function but more to the point, they provide a demonstration that the use of ERPs generally and, in this case, P300 specifically, can be used with confidence in nonresponsive populations in the knowledge that one is obtaining accurate and valuable psychometric information about that individual’s cognitive status.

Of course, context within a particular protocol is equally important. We have shown that the classic MMN oddball design of a frequently occurring series of “standard” tones interspersed with a less frequently occurring “deviant” tone results in larger MMN responses than an alternating pattern of tones (A,B) serving as the standard with a tone repetition (e.g., A,B,B,A,B) serving as the deviant. The P300 generated the strongest response when the subject’s own name was put together with tones, which are acoustically very different, rather than with other words and names, which are very similar in nature. The strongest N400 response was generated when the semantic violation was put in a sentence context rather than in a less semantically rich word-word priming environment. [49, 50]

The role of context strength is critically important when evaluating language comprehension generally and even more so when using ERPs in clinical environments. The choice of either a weaker word-word priming task instead of a stronger sentence design or the failure to ensure that sentences are structured for maximum contextual strength can result in healthy controls failing to show hypothesized N400 effects. This failure to establish a clear baseline control makes interpretation of patients’ failures to show N400 responses impossible to interpret. [51, 52]

When comparing the two cohorts of younger participants in their performance on the two N400 paradigms, we see a decline in the number of responding participants in the smaller, second cohort. However, the pattern of the decline in performance with the reduction in strength of context still holds, both in the passive and active conditions in the second cohort. In both behavioural conditions, the number of responding participants to the word-word priming paradigm are nearly half of those who responded to the semantic violation sentences. While there are paradigms that are able to elicit the N400 with even stronger contexts, such as those used by van Berkum et al. [53] that place the violation in the middle of the sentence or within a larger discourse, care must be taken to balance the cognitive processing requirements of those paradigms with their contextual strength. These paradigms may be too taxing on patients with traumatic brain injuries or diminished capacity, which may lead to false negatives or cases with too few good trials for averaging.

It is important to reiterate that the only instruction given to the participants in the first cohort was that they need not pay attention to the stimuli. As has been noted elsewhere (see [24] for a review of N400 and attention, and [54] for a discussion of the P300 and attention), not attending to the stimuli can significantly reduce the amplitude of an ERP response. As was seen in from the first cohort, the most robust responses were generated when the stimuli were salient and contextually different enough to attract attention regardless of whether the stimuli were being attended to explicitly or not. An enhancement of this effect was seen when the participants were instructed to pay attention to the stimuli.

These results demonstrate the effects of attention on ERPs and by implication the processes they reflect. They also show that these effects are altered by the aging process. For example, N400 amplitudes to semantic violations in sentences were observed in both the younger and older adult participants; however, N400 amplitudes were reduced by 50% in the older adults. This same pattern of age-related attenuation is seen in several other paradigms, like the P300 response to the oddball names, the pattern violation MMN, and the word-word priming. Given that at least 75% of strokes in Canada are in people over the age of 65 [55], and that coma is often a consequence of stroke, it is very likely that this age-related ERP degradation may become a constant background feature that should be acknowledged and accounted for in future work. Similarly, providing instructions to patients regardless of diagnosis and apparent state of consciousness as well as using the most stable paradigms available from the literature are all procedurally essential for providing patients the opportunity to generate the most robust responses of which they may capable.

In summary, we recommend the use of an auditory oddball paradigm that includes novel stimuli to elicit the MMN and P300, and semantic violation sentences to elicit the N400. As we have demonstrated and as illustrated in Table 11, on average, almost 90% of all participants showed a MMN to the auditory oddball paradigm, and nearly 100% of all participants showed a P300 to the novel stimuli in this paradigm. While the proportion of participants responding to the semantic violation sentences was markedly lower than the auditory oddball paradigm, the stronger context provided by the sentences improved the response rate compared to using a word-word priming paradigm. There was also an enhancing effect of paying attention to rather than ignoring the semantic violation sentences, with the number of participants showing a N400 doubling. This combination of paradigms appears to allow for a robust response in the absence of explicit attention, and are reinforced when the participant is instructed to pay attention. It is also important to note that when applying these tests to clinical environments, that the absence of a positive response should not be interpreted as a negative response. The purpose of these assessments, at least in their current state, should be to better inform clinicians and enable to start rehabilitation treatments earlier in cases of positive results.

## Supporting information

S1 Fig Grand average difference waveforms of the oddball mismatch MMN and corresponding peak topographic maps. The mean difference response for the younger adult (blue) and older adult (orange) groups are plotted for the (A) Mid Frontal, (B) Mid Central, and (C) Mid Parietal ROIs. Dashed colored lines indicate the mean group latency from individually scored MMN peak latencies. Scalp topography maps show voltage distributions at mean group peak latencies.
(TIF)

S2 Fig Grand average waveforms to the familiar and unfamiliar novels and corresponding peak topographic maps within the oddball mismatch. The mean responses to the familiar novel (FN) for younger adults (blue) and older adults (green), and the unfamiliar novel (UFN) for younger adults (orange) and older adults (red) groups are plotted for the (A) Mid Frontal, (B) Mid Central, and (C) Mid Parietal ROIs. Dashed colored lines indicate the mean group latency from individually scored P300 peak latencies. Scalp topography maps show voltage distributions at mean group peak latencies.
(TIF)

S3 Fig Grand average difference waveforms of the pattern violation mismatch MMN to the first and second deviants and corresponding peak topographic maps. The mean difference response to the first and second deviants for the young adult and older adult groups are plotted for the (A) Mid Frontal, (B) Mid Central, and (C) Mid Parietal ROIs. Young adult first deviant (blue), young adult second deviant (orange), older adult first deviant (green), and older adult second deviant (red). Dashed colored lines indicate the mean group latency from individually scored MMN peak latencies. Scalp topography maps show voltage distributions at mean group peak latencies.
(TIF)

S4 Fig Grand average waveforms at all ROIs to a list of Common First Names, the Subject’s Own Name, and a list of Non-salient Other Words and their corresponding peak topographic maps within the Subject’s Own Name paradigm. The younger adult group’s average responses in the (A) Mid Frontal, (C) Mid Central, (E) Mid Parietal ROIs, and the older adult group’s average responses in the (B) Mid Frontal, (D) Mid Central, (F) Mid Parietal ROIs to Common First Names (blue), Subject’s Own Name (orange), and the list of Non-salient Other Words (green). Dashed colored lines indicate the mean group latency from individually scored P300 peak latencies. Scalp topography maps show voltage distributions at mean group peak latencies.
(TIF)

S5 Fig Grand average waveforms at all ROIs to Congruent, Incongruent, Low Probability, and Phonological Foil terminal words and their corresponding peak topographic maps within the semantic violation sentences paradigm. The younger adult group’s average responses in the (A) Mid Frontal, (C) Mid Central, (E) Mid Parietal ROIs, and the older adult group’s average responses in the (B) Mid Frontal, (D) Mid Central, (F) Mid Parietal ROIs to Congruent (blue), Low Probability (orange), Incongruent (green) and Phonological Foil (red) terminal words are plotted. Dashed colored lines indicate the mean group latency from individually scored N400 peak latencies. Scalp topography maps show voltage distributions at mean group peak latencies.
(TIF)

S6 Fig Grand average waveforms at all ROIs to Congruent, Incongruent, Nonword, and Pseudoword target words and their corresponding peak topographic maps within the word-word priming paradigm. The younger adult group’s average responses in the (A) Mid Frontal, (C) Mid Central, (E) Mid Parietal ROIs, and the older adult group’s average responses in the (B) Mid Frontal, (D) Mid Central, (F) Mid Parietal ROIs to the list of Congruent (blue), Incongruent (orange), Nonword (green) and Pseudoword (red) target words are plotted. Dashed colored lines indicate the mean group latency from individually scored N400 peak latencies. Scalp topography maps show voltage distributions at mean group peak latencies.
(TIF)

S7 Fig Grand average waveforms at all ROIs to a list of Common First Names, the Subject’s Own Name, and a list of Non-salient Other Words and their corresponding peak topographic maps within the Subject’s Own Name paradigm with the behavioral manipulation. The active condition average responses in the (A) Mid Frontal, (C) Mid Central, (E) Mid Parietal ROIs, and the passive condition average responses in the (B) Mid Frontal, (D) Mid Central, (F) Mid Parietal ROIs to the the Common First Names (blue), Subject’s Own Name (orange), and the list of Non-salient Other Words (green) are plotted. Dashed colored lines indicate the mean group latency from individually scored P300 peak latencies. Scalp topography maps show voltage distributions at mean group peak latencies.
(TIF)

S8 Fig Grand average waveforms at all ROIs to Congruent, Incongruent, Low Probability, and Phonological Foil terminal words and their corresponding peak topographic maps within the semantic violation sentences paradigm with the behavioral manipulation. The active condition average responses in the (A) Mid Frontal, (C) Mid Central, (E) Mid Parietal ROIs, and the passive condition average responses in the (B) Mid Frontal, (D) Mid Central, (F) Mid Parietal ROIs to Congruent (blue), Low Probability (orange), Incongruent (green) and Phonological Foil (red) terminal words are plotted. Dashed colored lines indicate the mean group latency from individually scored N400 peak latencies. Scalp topography maps show voltage distributions at mean group peak latencies.
(TIF)

S9 Fig Grand average waveforms at all ROIs to Congruent, Incongruent, Nonword, and Pseudoword target words and their corresponding peak topographic maps within the word-word priming paradigm with the behavioral manipulation. The active condition average responses in the (A) Mid Frontal, (C) Mid Central, (E) Mid Parietal ROIs, and the passive condition average responses in the (B) Mid Frontal, (D) Mid Central, (F) Mid Parietal ROIs to the list of Congruent (blue), Incongruent (orange), Nonword (green) and Pseudoword (red) target words are plotted. Dashed colored lines indicate the mean group latency from individually scored N400 peak latencies. Scalp topography maps show voltage distributions at mean group peak latencies.
(TIF)
