# Supplementary figures and images for: A framework for the extended monitoring of levels of cognitive function in unresponsive patients
Source: PLoS One. 2018 Jul 19;13(7):e0200793. doi: 10.1371/journal.pone.0200793 (PMC6053194; doi:10.1371/journal.pone.0200793)

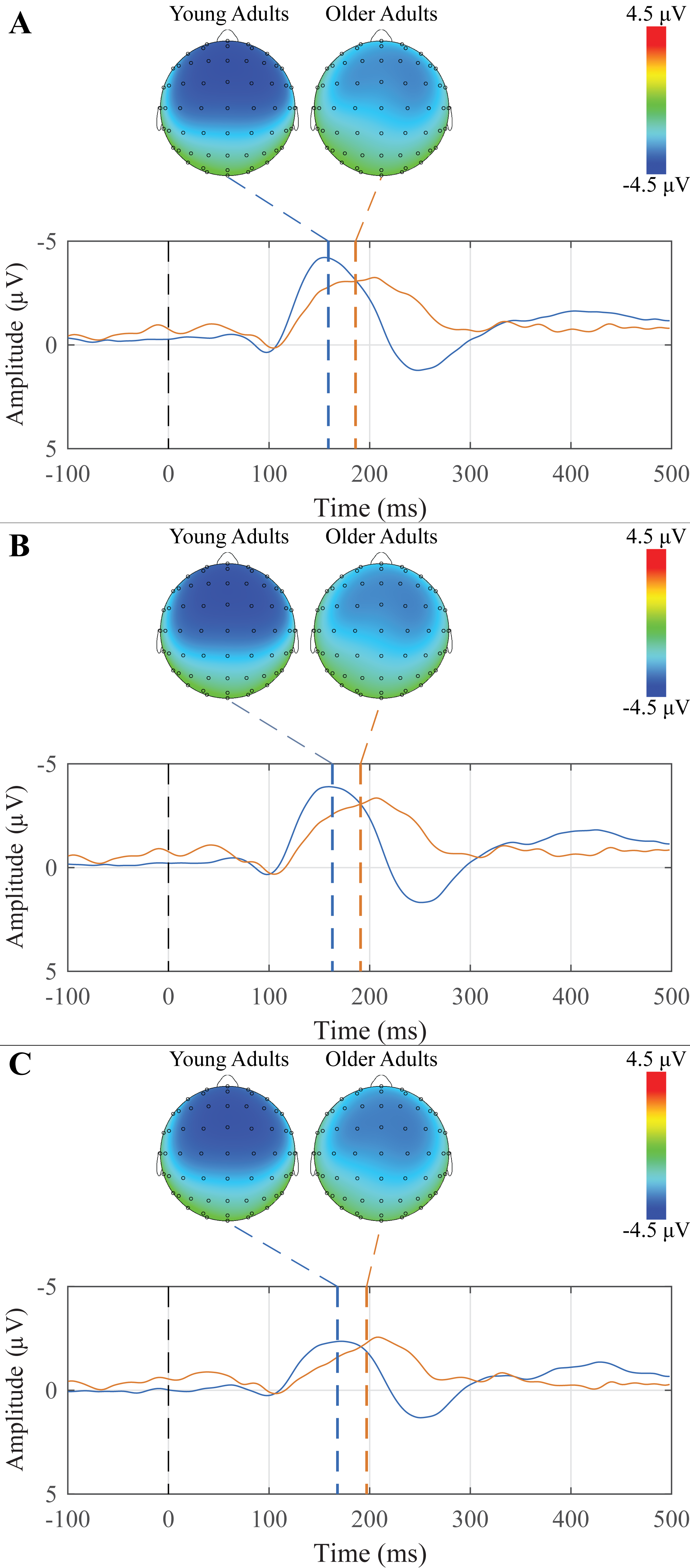

Supplement: S1 Fig — The mean difference response for the younger adult (blue) and older adult (orange) groups are plotted for the (A) Mid Frontal, (B) Mid Central, and (C) Mid Parietal ROIs. Dashed colored lines indicate the mean group latency from individually scored MMN peak latencies. Scalp topography maps show voltage distributions at mean group peak latencies. (TIF) [file pone.0200793.s001.tif]

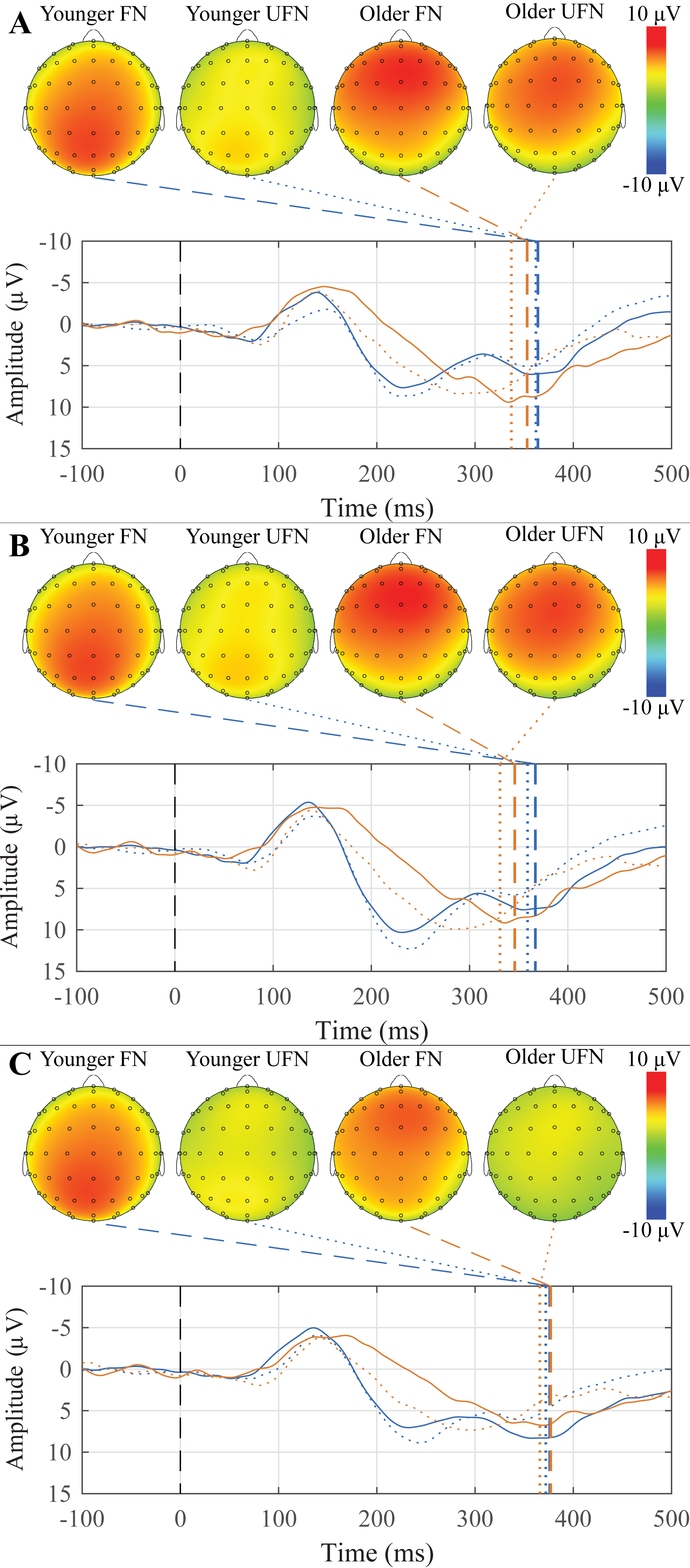

Supplement: S2 Fig — The mean responses to the familiar novel (FN) for younger adults (blue) and older adults (green), and the unfamiliar novel (UFN) for younger adults (orange) and older adults (red) groups are plotted for the (A) Mid Frontal, (B) Mid Central, and (C) Mid Parietal ROIs. Dashed colored lines indicate the mean group latency from individually scored P300 peak latencies. Scalp topography maps show voltage distributions at mean group peak latencies. (TIF) [file pone.0200793.s002.tif]

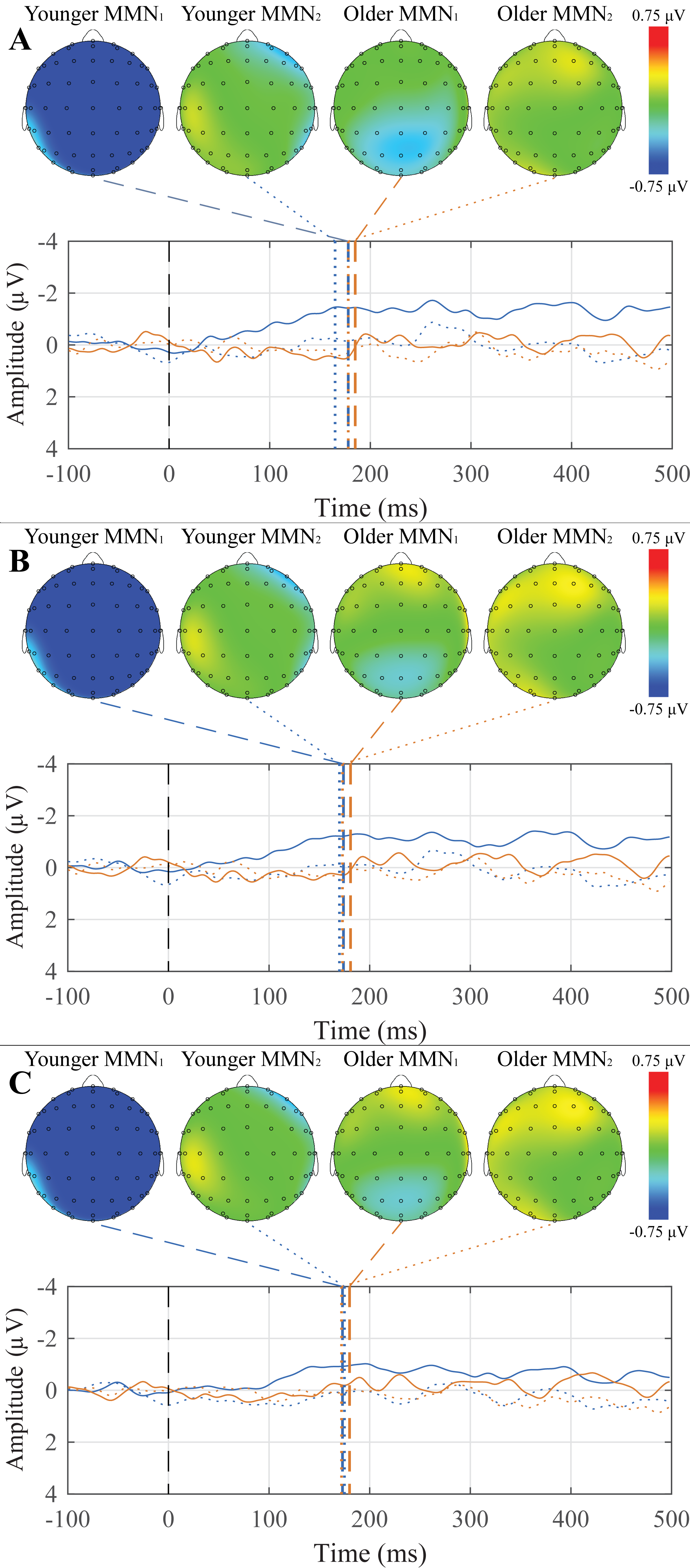

Supplement: S3 Fig — The mean difference response to the first and second deviants for the young adult and older adult groups are plotted for the (A) Mid Frontal, (B) Mid Central, and (C) Mid Parietal ROIs. Young adult first deviant (blue), young adult second deviant (orange), older adult first deviant (green), and older adult second deviant (red). Dashed colored lines indicate the mean group latency from individually scored MMN peak latencies. Scalp topography maps show voltage distributions at mean group peak latencies. (TIF) [file pone.0200793.s003.tif]

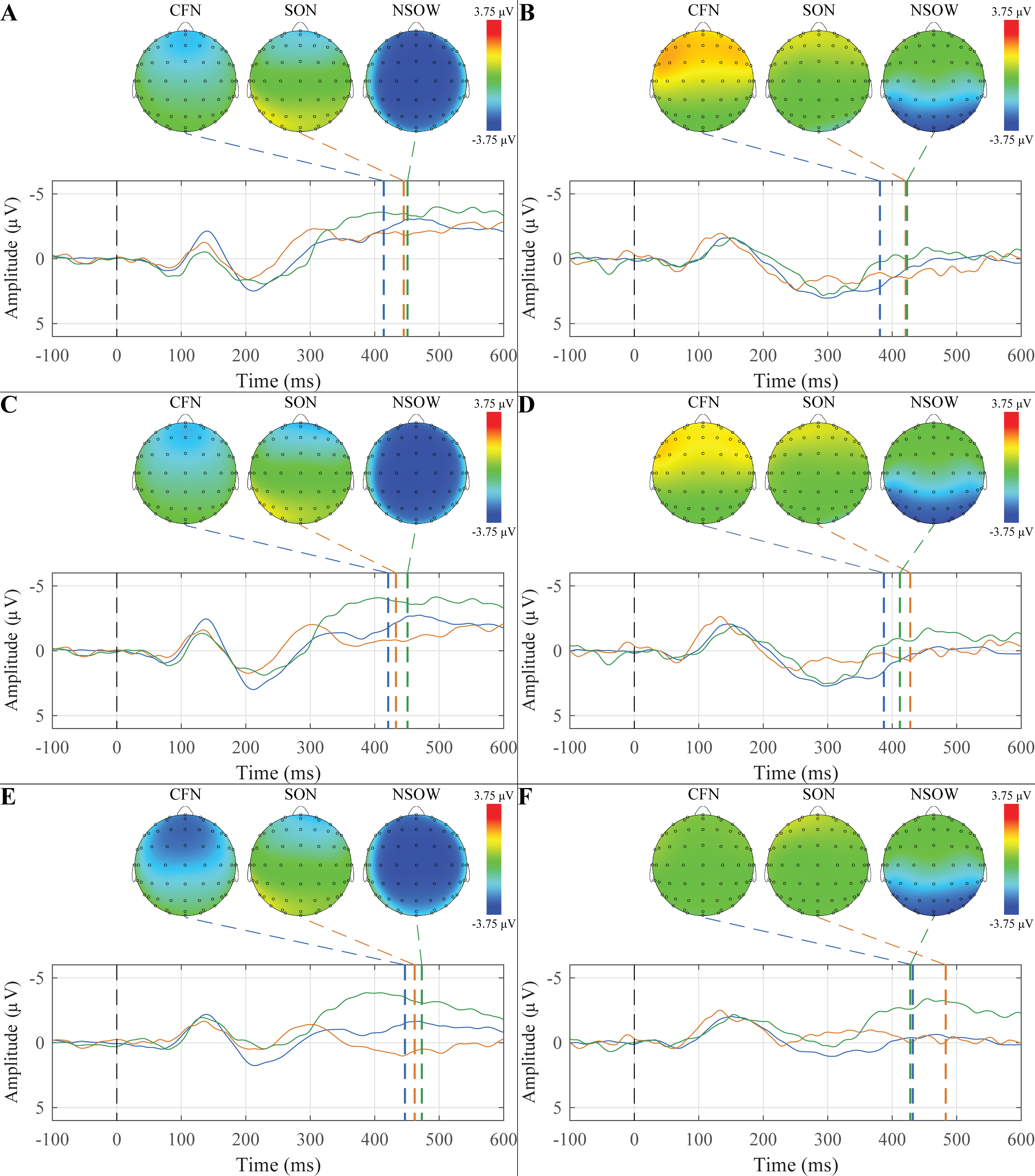

Supplement: S4 Fig — The younger adult group’s average responses in the (A) Mid Frontal, (C) Mid Central, (E) Mid Parietal ROIs, and the older adult group’s average responses in the (B) Mid Frontal, (D) Mid Central, (F) Mid Parietal ROIs to Common First Names (blue), Subject’s Own Name (orange), and the list of Non-salient Other Words (green). Dashed colored lines indicate the mean group latency from individually scored P300 peak latencies. Scalp topography maps show voltage distributions at mean group peak latencies. (TIF) [file pone.0200793.s004.tif]

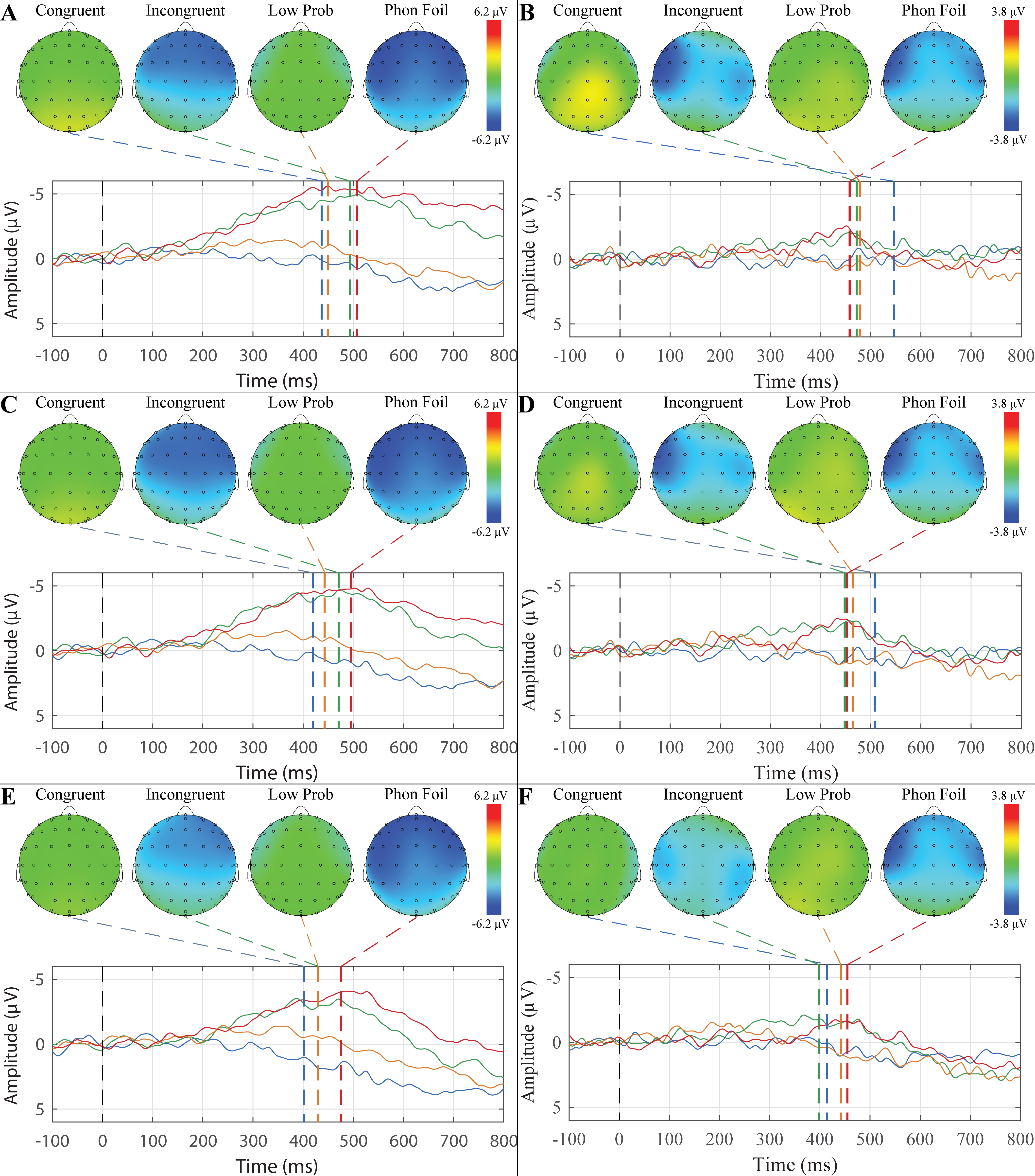

Supplement: S5 Fig — The younger adult group’s average responses in the (A) Mid Frontal, (C) Mid Central, (E) Mid Parietal ROIs, and the older adult group’s average responses in the (B) Mid Frontal, (D) Mid Central, (F) Mid Parietal ROIs to Congruent (blue), Low Probability (orange), Incongruent (green) and Phonological Foil (red) terminal words are plotted. Dashed colored lines indicate the mean group latency from individually scored N400 peak latencies. Scalp topography maps show voltage distributions at mean group peak latencies. (TIF) [file pone.0200793.s005.tif]

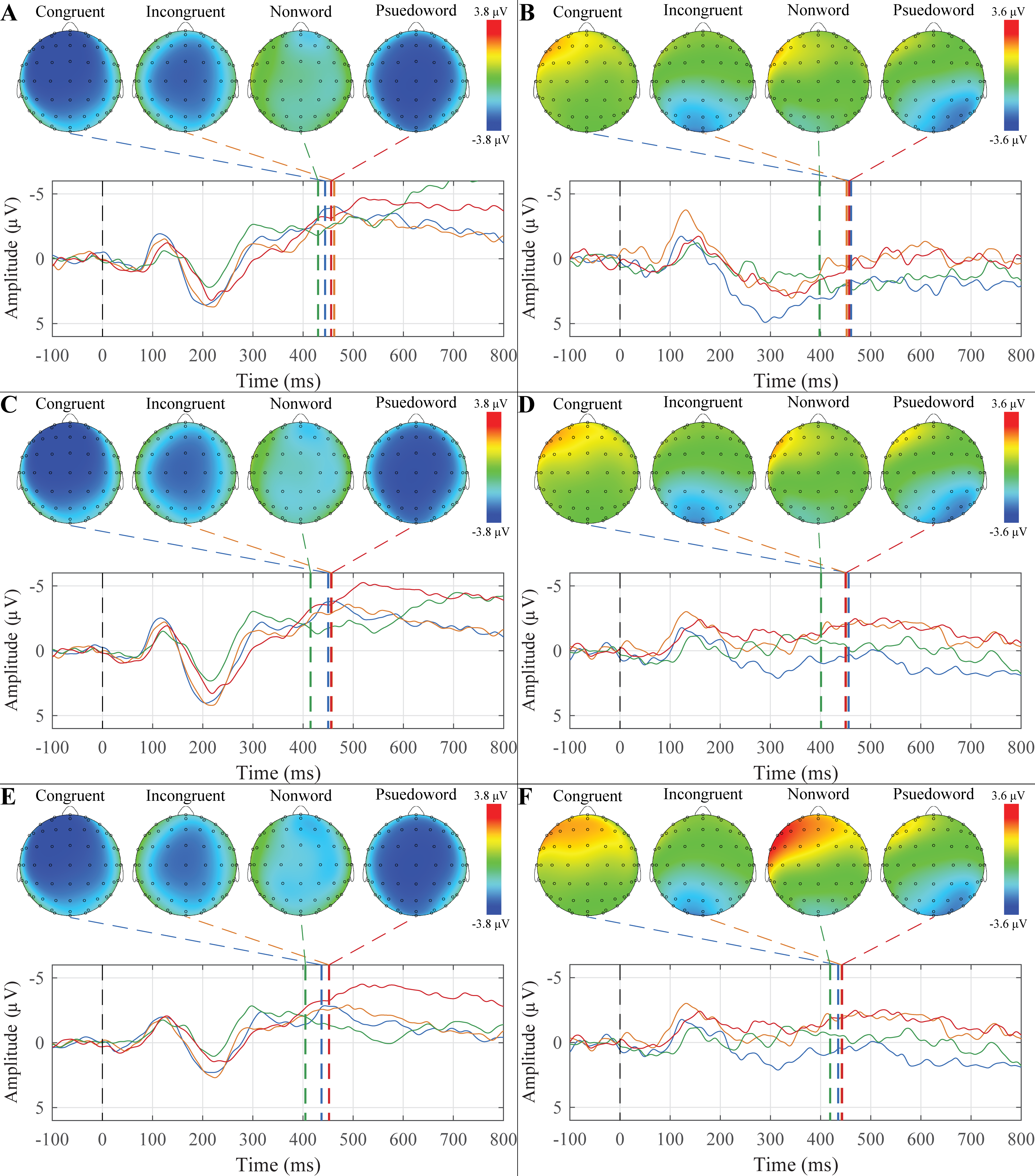

Supplement: S6 Fig — The younger adult group’s average responses in the (A) Mid Frontal, (C) Mid Central, (E) Mid Parietal ROIs, and the older adult group’s average responses in the (B) Mid Frontal, (D) Mid Central, (F) Mid Parietal ROIs to the list of Congruent (blue), Incongruent (orange), Nonword (green) and Pseudoword (red) target words are plotted. Dashed colored lines indicate the mean group latency from individually scored N400 peak latencies. Scalp topography maps show voltage distributions at mean group peak latencies. (TIF) [file pone.0200793.s006.tif]

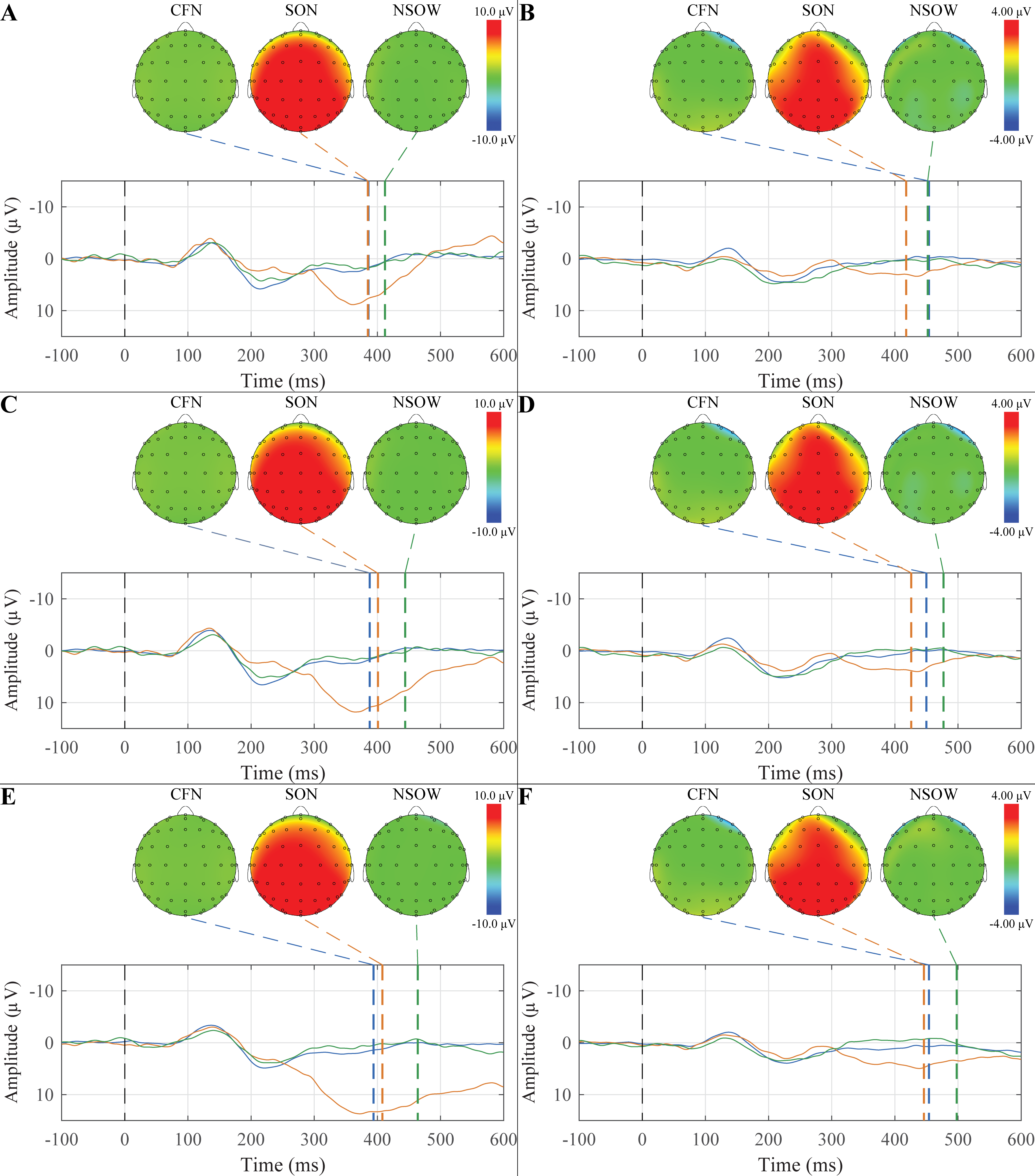

Supplement: S7 Fig — The active condition average responses in the (A) Mid Frontal, (C) Mid Central, (E) Mid Parietal ROIs, and the passive condition average responses in the (B) Mid Frontal, (D) Mid Central, (F) Mid Parietal ROIs to the the Common First Names (blue), Subject’s Own Name (orange), and the list of Non-salient Other Words (green) are plotted. Dashed colored lines indicate the mean group latency from individually scored P300 peak latencies. Scalp topography maps show voltage distributions at mean group peak latencies. (TIF) [file pone.0200793.s007.tif]

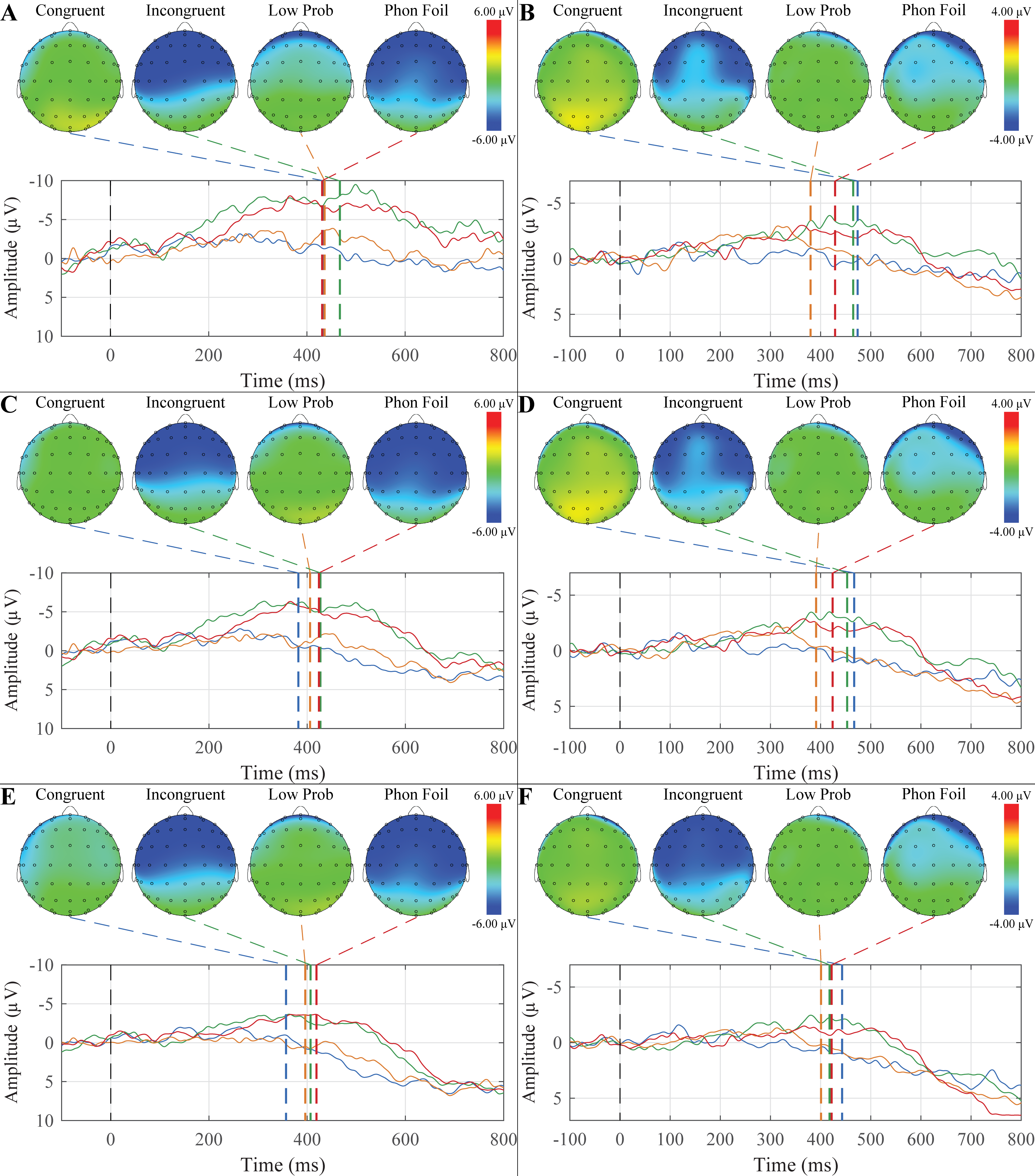

Supplement: S8 Fig — The active condition average responses in the (A) Mid Frontal, (C) Mid Central, (E) Mid Parietal ROIs, and the passive condition average responses in the (B) Mid Frontal, (D) Mid Central, (F) Mid Parietal ROIs to Congruent (blue), Low Probability (orange), Incongruent (green) and Phonological Foil (red) terminal words are plotted. Dashed colored lines indicate the mean group latency from individually scored N400 peak latencies. Scalp topography maps show voltage distributions at mean group peak latencies. (TIF) [file pone.0200793.s008.tif]

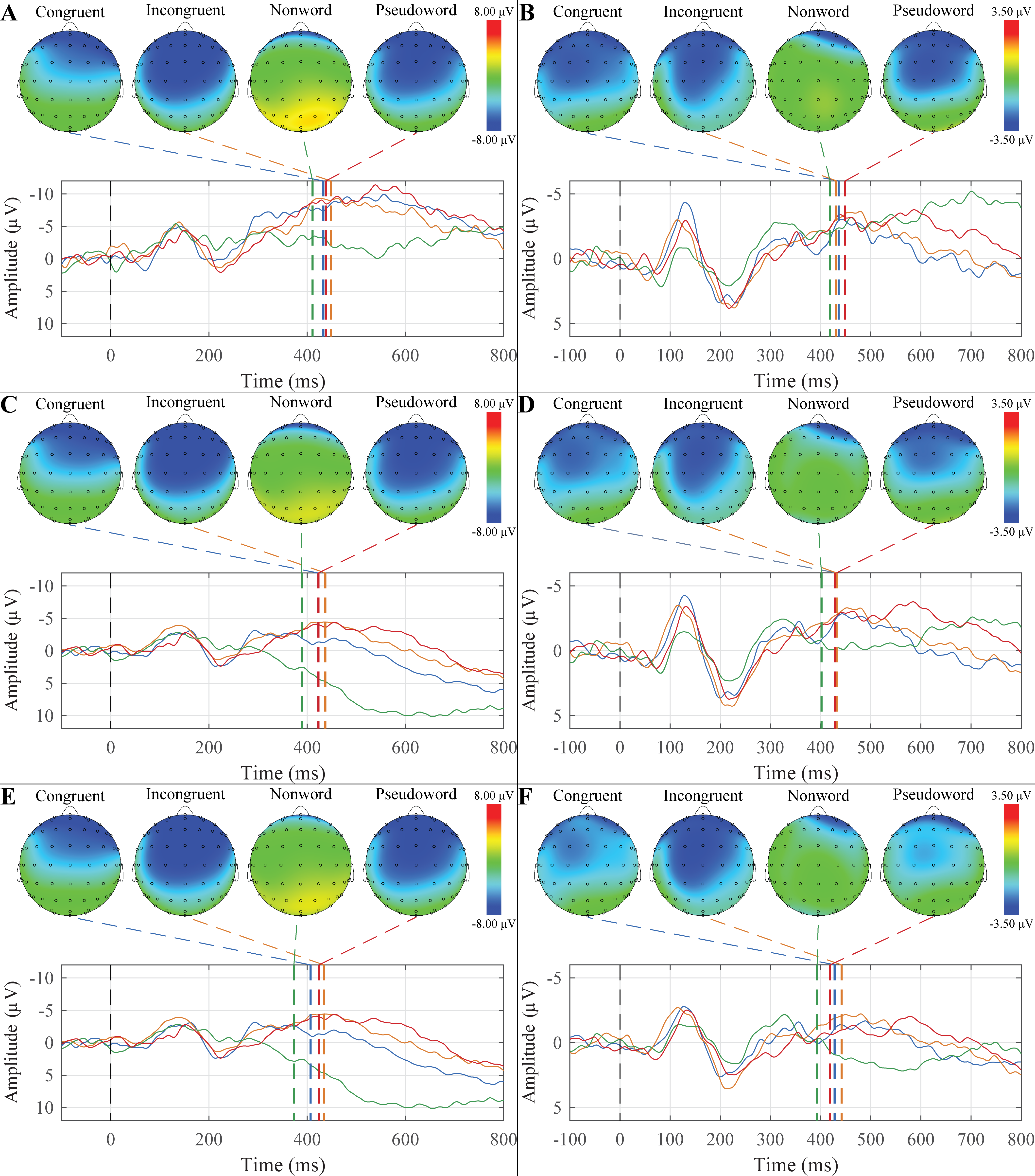

Supplement: S9 Fig — The active condition average responses in the (A) Mid Frontal, (C) Mid Central, (E) Mid Parietal ROIs, and the passive condition average responses in the (B) Mid Frontal, (D) Mid Central, (F) Mid Parietal ROIs to the list of Congruent (blue), Incongruent (orange), Nonword (green) and Pseudoword (red) target words are plotted. Dashed colored lines indicate the mean group latency from individually scored N400 peak latencies. Scalp topography maps show voltage distributions at mean group peak latencies. (TIF) [file pone.0200793.s009.tif]
